# Higher-order lattice anharmonicity reshaping non-equilibrium carrier dynamics in wide-phonon-gap semiconductors

**DOI:** 10.1093/nsr/nwag224

**Published:** 2026-04-20

**Authors:** Te-Huan Liu, Xin Qian, Shuai Yue, Ming Li, Zhichun Liu, Bai Song, Fei Tian, Xinfeng Liu, Ronggui Yang

**Affiliations:** School of Energy and Power Engineering, Huazhong University of Science and Technology, Wuhan 430074, China; School of Energy and Power Engineering, Huazhong University of Science and Technology, Wuhan 430074, China; Chinese Academy of Sciences Key Laboratory of Standardization and Measurement for Nanotechnology, National Center for Nanoscience and Technology, Beijing 100190, China; School of Energy and Power Engineering, Huazhong University of Science and Technology, Wuhan 430074, China; School of Energy and Power Engineering, Huazhong University of Science and Technology, Wuhan 430074, China; College of Mechanics and Engineering Science, Peking University, Beijing 100871, China; School of Materials Science and Engineering, Sun Yat-sen University, Guangzhou 510006, China; Chinese Academy of Sciences Key Laboratory of Standardization and Measurement for Nanotechnology, National Center for Nanoscience and Technology, Beijing 100190, China; College of Mechanics and Engineering Science, Peking University, Beijing 100871, China

**Keywords:** hot-phonon delocalization, carrier thermalization, four-phonon coupling, phonon bottleneck, boron-group V semiconductors

## Abstract

In semiconductors where three-phonon decay channels are suppressed, a mechanism termed “hot-phonon delocalization” induced by higher-order anharmonic decays emerges as the dominant process governing carrier thermalization. By combining *ab initio* solutions of the time-dependent coupled electron-phonon Boltzmann transport equations with femtosecond stimulated Raman spectroscopy, this study demonstrates that four-phonon couplings can substantially reshape the non-equilibrium carrier dynamics in semiconductors with significant phonon gaps, such as BAs and BSb. The results show that momentum-redistribution channels, specifically *o*+*o*→*o*+*o* and *o*+*a*→*o*+*a*, effectively delocalize hot phonons that initially accumulate in long-wavelength states by spreading them across the Brillouin zone. This behavior is fundamentally different from the conventional picture, where hot-phonon relaxation is assumed to be governed primarily by emission processes. This mechanism suppresses hot-phonon accumulation and enhances the efficiency of three-phonon Ridley- and Vallée-Bogani-type decays, mitigating the phonon bottleneck effect and ultimately improving Joule heating efficiency. In BAs, four-phonon coupling increases the energy dissipation rate of optical phonons nearly 70-fold, reducing phonon reabsorption and enabling high-energy electrons and holes to continuously emit optical phonons, as supported by our proposed phenomenological model. Consequently, the onset of phonon reabsorption heating is delayed from 6.0 to 12.9 ps. These findings provide a comprehensive understanding of carrier thermalization, revealing that hot-phonon delocalization governs energy-exchange pathways in wide-phonon-gap semiconductors on the picosecond timescale.

## Teaser

This study reveals that four-phonon coupling can spread hot lattice vibrations across momentum space, helping high-energy charge carriers release energy and thermalize more efficiently.

## INTRODUCTION

The dynamics of energy carriers in non-equilibrium states govern a wide range of intriguing phenomena in modern electronic devices [1–5]. For instance, carriers with excess energy can exploit their high ballistic transport velocities, enabling ultrafast device operation and reduced thermal dissipation in electronic chips [6]. Recent studies indicate that carrier multiplication through heterostructure engineering not only induces negative differential resistance but also reduces the subthreshold swing below the Boltzmann limit of 60 mV/dec in hot-emitter transistors [7]. In photovoltaics and photocatalysis, harnessing hot carriers enhances quantum efficiency and may potentially surpass the Shockley-Queisser limit [8,9]. However, high-energy carriers are not always beneficial. A notable example is hot carrier injection, typically considered a parasitic effect in transistors, generating excessive electron-hole pairs. Under gate bias, these energetic charge carriers may become trapped in the gate oxide or contribute to substrate leakage current, compromising device reliability [10–12]. Such non-equilibrium processes, whether advantageous or detrimental, are strongly affected by the distribution and lifetime of hot carriers.

The lifespan of hot carriers unfolds through a series of ultrafast processes [13]. Upon excitation by an external electric field or ultrafast laser heating, a fraction of charge carriers is driven into non-thermal energy states. Within the initial ∼10 fs, these carriers rapidly thermalize within the electronic subsystem through statically screened Coulomb interactions, Auger recombination, and impact ionization [14–16]. Thereafter, energy is predominantly transferred from the electrons to optical phonons in ∼100 fs, resulting in the generation of hot phonons [17,18]. After this stage, the electronic distribution can be well approximated by the Fermi-Dirac distribution at elevated temperatures, as electrons have thermalized near the band extrema [19,20]. Beyond ∼1 ps, in addition to ongoing electron-phonon (e-ph) coupling, the excited hot phonons relax within the lattice through phonon-phonon (ph-ph) scattering. Here, e-ph interactions dictate the evolution of electron temperature on timescales up to sub-microseconds, while ph-ph scattering serves as the dominant relaxation pathway for non-thermal lattice vibrations, which, in turn, affect electron temperature by reshaping the phonon distribution. In some materials, the internal thermalization timescale of the electronic and lattice subsystems can be comparable to, or even exceed, that of e-ph coupling. Under such conditions, the carrier distribution may exhibit strong anisotropy in momentum space [21–25]. Additionally, when the e-ph matrix elements are highly selective, as in polar semiconductors, localized thermal reservoirs can form within the crystal, giving rise to the phonon bottleneck effect [26,27]. These transport characteristics disrupt the homogeneity of particle interactions and invalidate the single-temperature approximation. Consequently, a time-dependent, mode-resolved treatment of carrier eigenstate evolution [28–30], or the solution of occupation functions through coupled e-ph transport equations [19,20,25,31–40], becomes necessary to capture the temporal and spatial variations of hot phonons.

Conventional understanding has long held that thermal carrier relaxation occurs primarily through three-phonon interactions. Four major three-phonon mechanisms—namely, the Klemens [41], Vallée-Bogani [42], Ridley [43], and Barman-Srivastava decays [44]—have been widely regarded as the principal channels for dissipating excess energy carried by longitudinal optical (LO) phonons. However, this assumption becomes questionable in semiconductors where four-phonon processes significantly affect carrier transport, or where transverse optical (TO) phonons are non-negligible. Previous studies have shown that four-phonon-mediated thermal resistance can dominate heat conduction in cubic BAs [45–49] and many other materials [50–57]. In BAs, this behavior arises from its exceptionally large acoustic-optical (*a*-*o*) energy gap (∼40 meV), stemming from the mass disparity between its constituent atomic species, which suppresses conventional three-phonon processes [58]. Ultrafast electron microscopy experiments have reported unusually long-lived populations (∼200 ps) of photoexcited electrons and holes in BAs [59]. These extended ambipolar diffusion lengths have been attributed to a phonon bottleneck induced by the large *a*-*o* gap. Using first-principles calculations, Sadasivam *et al.* [32] also demonstrated that, unlike in silicon and other materials with insignificant phonon gaps, the electronic and lattice subsystems in BAs remain out of thermal equilibrium for timescales exceeding 25 ps. These observations underscore several fundamental, yet unresolved issues, following a logical progression as follows. Does four-phonon coupling essentially reshape the phonon distribution and charge-carrier temperature in semiconductors with wide *a*-*o* gaps? If so, what are the dominant four-phonon-mediated decay channels within the lattice subsystem? More importantly, can four-phonon processes surpass conventional three-phonon processes, such as Ridley decay, or do they instead act to facilitate these processes in mitigating the hot-phonon bottleneck?

## RESULTS AND DISCUSSION

### Modeling of four-phonon coupling in carrier thermalization

To address these issues, the dynamics of local electron and phonon thermalization were investigated by solving time-dependent occupation functions governed by coupled Boltzmann transport equations (BTEs), which incorporate e-ph, phonon-electron (ph-e), and ph-ph interactions [60]:


(1) \begin{eqnarray*}
\frac{{d{f}_{n{{\bf k}}}\!\left( t \right)}}{{dt}} = {\left. {\frac{{\partial {f}_{n{{\bf k}}}\! \left( t \right)}}{{\partial t}}} \right|}_{{\mathrm{e \hbox{-} ph}}}{\mathrm{,}}
\end{eqnarray*}

(2) \begin{eqnarray*}
&&\frac{{d{n}_{\nu {{\bf q}}}\left( t \right)}}{{dt}}\\ &&= {\left. {\frac{{\partial {n}_{\nu {{\bf q}}}\left( t \right)}}{{\partial t}}} \right|}_{{\mathrm{ph \hbox{-} e}}} + {\left. {\frac{{\partial {n}_{\nu {{\bf q}}}\left( t \right)}}{{\partial t}}} \right|}_{{\mathrm{3 \hbox{-} ph}}} + {\left. {\frac{{\partial {n}_{\nu {{\bf q}}}\left( t \right)}}{{\partial t}}} \right|}_{{\mathrm{4 \hbox{-} ph}}}{\mathrm{.}}\\
\end{eqnarray*}

Here, ${f}_{n{{\bf k}}}( t )$ and ${n}_{\nu {{\bf q}}}( t )\ $represent the time-dependent distribution functions of electrons and phonons, respectively. The subscripts *n* and **k** refer to the electronic band index and wavevector, while *ν* and **q** are used for phonons. Our analysis focuses on the transient regime from 0.1 to 30 ps, during which e-ph and ph-ph interactions dominate energy exchange, and thermal diffusion is negligible. Consequently, electron-electron Coulomb scattering and drift terms are excluded. The principal advancement of this work lies in explicitly incorporating four-phonon vertex contributions, represented by the final term in Eq. (2). Due to the rapid thermalization of electrons (typically within a few femtoseconds), the electron population can be well approximated by a time-dependent Fermi-Dirac distribution $f_{n{{\bf k}}}^{\ {\mathrm{0}}}( t )$ at an elevated electron temperature. Accordingly, the time derivative of the electron distribution is expressed as: ${\varepsilon }_{n{{\bf k}}}df_{n{{\bf k}}}^{\ {\mathrm{0}}}/dt = {C}_{\mathrm{e}}d{T}_{\mathrm{e}}/dt$, where ${C}_{\mathrm{e}}$ and ${T}_{\mathrm{e}}$ are, respectively, the instantaneous electronic heat capacity and electron temperature, and ${\varepsilon }_{n{{\bf k}}}$ is the electron energy relative to the Fermi level. Using this formulation, Eq. (1) can be solved from the phonon perspective through ph-e collisions, provided that energy conservation between the electronic and lattice subsystems must be guaranteed: $N_{{\bf k}}^{\ - {\mathrm{1}}}\mathop \sum \nolimits_{n{\mathrm{,\ }}{{\bf k}}} {\varepsilon }_{n{{\bf k}}}d{f}_{n{{\bf k}}}/dt = - N_{{\bf q}}^{\ - {\mathrm{1}}}\mathop \sum \nolimits_{\nu {\mathrm{,\ }}{{\bf q}}} \hbar {\omega }_{\nu {{\bf q}}}\partial {n}_{\nu {{\bf q}}}/\partial t{|}_{{\mathrm{ph \hbox{-} e}}}$, where ${N}_{{\bf k}}$ and ${N}_{{\bf q}}$ denote the number of discrete **k** and **q** points in the Brillouin zone, and $\hbar {\omega }_{\nu {{\bf q}}}$ is the phonon energy. This equation specifies that the energy lost by electrons is instantaneously transferred to the lattice, which defines the energy transfer rate ${Q}_{{\mathrm{e \hbox{-} ph}}}( t )$. Using a first-order time-stepping scheme, Eqs (1) and (2) can be recast in recursive form:


(3) \begin{eqnarray*}
{T}_{\mathrm{e}}\left( {t{\mathrm{ + \Delta }}t} \right) &=& {T}_{\mathrm{e}}\left( t \right)\\
&&- \frac{{{\mathrm{\Delta }}t}}{{{C}_{\mathrm{e}}\left( t \right)}}\left[ {\frac{{\mathrm{1}}}{{{N}_{{\bf q}}}}\mathop \sum \limits_{\nu {\mathrm{,}}{{\bf q}}} \hbar {\omega }_{\nu {{\bf q}}}{{\left. {\frac{{\partial {n}_{\nu {{\bf q}}}\left( t \right)}}{{\partial t}}} \right|}}_{{\mathrm{ph \hbox{-} e}}}} \right]{\mathrm{,}}\\
\end{eqnarray*}

(4) \begin{eqnarray*}
{n}_{\nu {{\bf q}}}\left( {t{\mathrm{ + \Delta }}t} \right) &=& {n}_{\nu {{\bf q}}}\left( t \right) + {\mathrm{ \Delta }}t\Bigg[ {{\left. {\frac{{\partial {n}_{\nu {{\bf q}}}\left( t \right)}}{{\partial t}}} \right|}}_{{\mathrm{ph \hbox{-} e}}}\\
&&+\, {{\left. {\frac{{\partial {n}_{\nu {{\bf q}}}\left( t \right)}}{{\partial t}}} \right|}}_{{\mathrm{3 - ph}}} + {{\left. {\frac{{\partial {n}_{\nu {{\bf q}}}\left( t \right)}}{{\partial t}}} \right|}}_{{\mathrm{4 \hbox{-} ph}}} \Bigg]{\mathrm{.}}\\
\end{eqnarray*}

These equations describe the time evolution of the electron temperature and mode-resolved phonon occupations, naturally incorporating successive thermalization effects in semiconductors with heterogeneous e-ph interactions and large phonon band gaps [32]. Explicit expressions for all collision integrals are provided in Note S1, and details of the *ab initio* simulations are provided in the Methods section and in Note S2.

### Hot-phonon delocalization and charge-carrier temperatures

**Figure 1.**
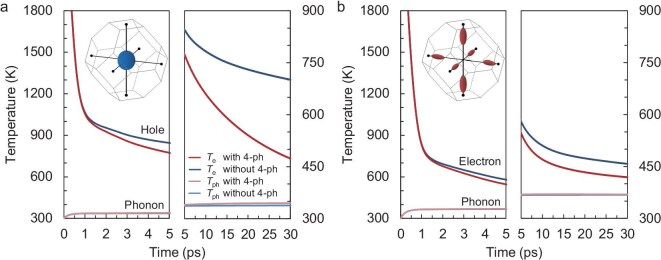
Time evolution of charge-carrier and phonon temperatures in BAs. (a) *p*-type conduction; (b) *n*-type conduction. Note that phonons remain out of equilibrium during thermalization; the phonon temperatures are ‘effective’ and are extracted by fitting the non-equilibrium distributions to an equivalent Bose-Einstein distribution for ease of interpretation.

Carrier thermalization dynamics were investigated in four cubic boron-group V semiconductors: BN, BP, BAs, and BSb. The Fermi level was set 0.15 eV above the conduction band minimum and below the valence band maximum to simulate *n*-type and *p*-type conduction, respectively. Figure 1 shows the time evolution of charge-carrier and phonon temperatures in BAs, initialized with an electronic temperature of 3000 K and a lattice temperature of 300 K [see panels (a) and (b) in Figs S4–S7 for other boron-group V semiconductors, and Movie S1 for the time evolution of mode-resolved phonon temperatures]. The results indicate that: (ⅰ) four-phonon coupling affects carrier thermalization after ∼1 ps, a characteristic timescale at which lattice thermal relaxation becomes active, and can reduce hole temperature by more than 200 K at 30 ps; (ⅱ) this effect is less pronounced for electrons, as the conduction band minima in zinc-blende solids are 6-fold degenerate, allowing thermalization through intervalley e-ph scatterings; (ⅲ) four-phonon-assisted thermalization of high-energy charge carriers is observed in BAs and BSb, but remains negligible in BN and BP. These findings indicate that four-phonon coupling facilitates energy dissipation from charge carriers to phonons in semiconductors with significant *a*-*o* gaps, in addition to its known role in limiting thermal conductivity in BAs. Notably, long-lived hot charge carriers persist in BAs and BSb, as the electronic and lattice subsystems do not fully relax within the timescale studied (see Fig. S24 for a longer simulation time), which is in qualitative agreement with the experimental observations [59]. Since four-phonon coupling acts exclusively within the lattice, its contribution to thermalization arises from enhancing the probability of momentum transfer from long-wavelength hot phonons to less-occupied optical modes, an effect referred to here as “hot-phonon delocalization” (see Movie S2 for visualization).

This four-phonon-induced delocalization enables high-energy charge carriers to deposit energy into the lattice more efficiently. To support this finding, we follow the analysis by Choudhry *et al.* [59] and propose a phenomenological model for the net absorption rate of a long-wavelength optical phonon by an electron at state *n***k** of a parabolic band through Fröhlich and optical-deformation-potential couplings (see Note S3 for derivation details):


(5) \begin{eqnarray*}
\Delta \mathcal{P}_{{{k}} + {{q}} \to {{k^{\prime}}}}^{{\mathrm{Fr\ddot{o}}}} &=& \mathcal{F}\Delta {{{n}}}_{{q}}\frac{{{\mathrm{\pi }}{{{e}}}^{\mathrm{2}}{{\mathrm{\omega }}}_{{q}}}}{{{\mathrm{\Omega }}{{{q}}}^{\mathrm{2}}}} \left( {\varepsilon _\infty ^{ - {\mathrm{1}}} - \varepsilon _{\mathrm{0}}^{ - {\mathrm{1}}}} \right) {\mathrm{\delta }}\\
&&\left( {{{\mathrm{\varepsilon }}}_{{k}} - {{\mathrm{\varepsilon }}}_{{{k^{\prime}}}} + \hbar {{\mathrm{\omega }}}_{{q}}} \right){{\mathrm{\delta }}}_{{{k}} + {{q,k^{\prime}}}}{\mathrm{,}}
\end{eqnarray*}

(6) \begin{eqnarray*}
\Delta \mathcal{P}_{{{k}} + {{q}} \to {{k^{\prime}}}}^{{\mathrm{ODP}}} = \mathcal{F}\Delta {{{n}}}_{{q}}\frac{{{\mathrm{\pi }}{{\mathrm{\Xi }}}^{\mathrm{2}}}}{{{\mathrm{\rho \Omega }}{{\mathrm{\omega }}}_{{q}}}}{\mathrm{\delta }}\left( {{{\mathrm{\varepsilon }}}_{{k}} - {{\mathrm{\varepsilon }}}_{{{k^{\prime}}}} + \hbar {{\mathrm{\omega }}}_{{q}}} \right){{\mathrm{\delta }}}_{{{k}} + {{q,k^{\prime}}}}{\mathrm{,}}
\end{eqnarray*}

where Ω is the volume of the unit cell, *e* is the elementary charge, *ρ* is the mass density, Ξ is the optical-deformation potential, and *ε*_0_ and *ε*_∞_ are the static and high-frequency dielectric parameters, respectively. The dimensionless prefactor $\mathcal{F}$ is defined as ${{{n}}}_{{e}}{( {\frac{{{\mathrm{2\pi }}{\hbar }^{\mathrm{2}}}}{{{{{m}}}_{{e}}{{{k}}}_{{B}}{{{T}}}_{{e}}}}} )}^{{\mathrm{3}}/{\mathrm{2}}}{\mathrm{exp}}( { - \frac{{{\hbar }^{\mathrm{2}}{{{k}}}^{\mathrm{2}}}}{{{\mathrm{2}}{{{m}}}_{{e}}{{{k}}}_{{B}}{{{T}}}_{{e}}}}} )$, which depends on the charge concentration (*n*_e_), effective electron mass (*m*_e_), and electron temperature. The equations indicate that the net phonon absorption rate, ${\mathrm{\Delta }}{\mathcal{P}}_{k + q\rightarrow k^{\prime}}$, remains small at very high electron temperatures, with energy exchange dominated by phonon emission. Phonon absorption becomes significant only after the charge carriers have cooled. This timescale approximately coincides with the onset of lattice thermal relaxation, by which point a substantial non-equilibrium phonon population has developed. In this framework, ${\mathrm{\Delta }}{n}_q$ is treated as a functional of the phonon-related coupling matrices. Therefore, if four-phonon scattering effectively facilitates the relaxation of long-wavelength LO phonons toward thermal equilibrium, as observed in BAs and BSb, the phonon absorption probability is suppressed, thereby mitigating the phonon bottleneck. This also demonstrates that four-phonon scattering can affect the charge-carrier temperature when higher-order anharmonic decays play an important role in carrier thermalization.

### Dominant anharmonic decay channels

**Figure 2.**
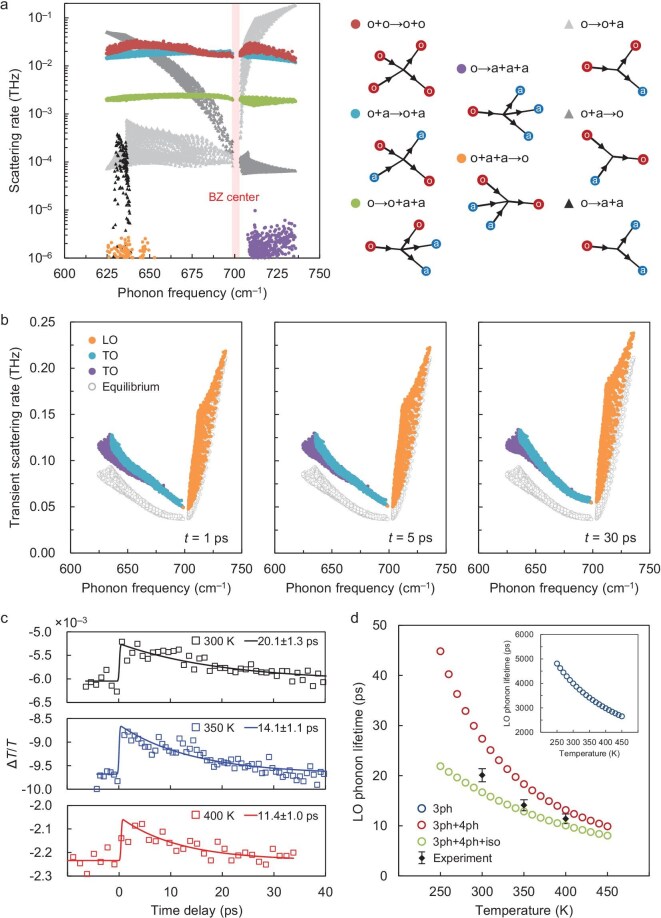
Optical-phonon scattering rates and lifetimes in *p*-type BAs. (a) Process-wise scattering rates of optical phonons at 300 K. Triangles and circles indicate three- and four-phonon processes, respectively. Schematic illustrations are shown on the right. (b) Transient scattering rates of optical phonons at *t* = 1, 5, and 30 ps. Open gray circles represent the equilibrium scattering rate evaluated at the initial phonon temperature of 300 K. (c) Phonon dynamics at 300, 350, and 400 K measured using FSRS. (d) Experimental and *ab initio* LO-phonon lifetimes over the temperature range from 250 to 450 K. Different colors denote different scattering mechanisms included in the simulations; black symbols represent the FSRS measurements.

Beyond the effectiveness of four-phonon interactions, identifying the dominant scattering channels responsible for the ultrafast thermalization of hot carriers is another critical issue. Given this, the process-wise ph-ph scattering rates for optical modes in BAs were analyzed, with schematic illustrations shown in Fig. 2a. Processes not shown are forbidden by symmetry selection rules or by the large *a*-*o* gap. Here, the equilibrium scattering rates evaluated at the initial phonon temperature of 300 K serve as a reference that quantifies the intrinsic strength of the ph-ph coupling kernels and the availability of allowed phase space under a common, well-defined condition. The *o*+*a*→*o*+*a* and *o*+*o*→*o*+*o* processes (blue and red symbols) represent anharmonic redistribution, in which an optical phonon absorbs an acoustic (or optical) phonon and converts into another optical and acoustic (or optical) phonon. The two redistribution channels show comparable scattering rates, with the former ranging from 1.3 × 10^−2^ to 2.2 × 10^−2^ THz and the latter from 1.3 × 10^−2^ to 3.6 × 10^−2^ THz. Their higher rates arise from the expanded scattering phase space associated with numerous quasiparticle combinations and from intrinsically stronger coupling among high-frequency vibrations. By contrast, the *o*→*o*+*a*+*a* and *o*→*a*+*a* + *a* processes (green and purple symbols), corresponding to direct decay of an optical phonon, require the participation of either two phonons with very low frequencies or three acoustic phonons, which substantially restricts the scattering probability because of the low phonon density of states in this frequency range. Both the emission processes show much lower scattering rates, with the former ranging from 1.4 × 10^−3^ to 2.5 × 10^−3^ THz and the latter below 1.0 × 10^−5^ THz. The transient scattering rates for optical phonons are also calculated as shown in Fig. 2b. Compared with the equilibrium scattering rates at 300 K (gray open symbols), the scattering rates increase with time owing to the growth in optical-phonon occupations by Joule heating. The overall contour of the scattering rates does not change remarkably during the thermalization, which supports the effectiveness of the use of the equilibrium scattering rate for analyzing the dominant phonon decay channels.

To assess the direct impact of each channel on thermalization dynamics, process-wise exclusion was performed in simulations of hole thermalization in BAs and BSb to clarify the specific role of each four-phonon process (Fig. S8a and c). The simulations indicate that hole-temperature reduction due to four-phonon coupling is dominated by the two redistribution channels: the *o*+*o*→*o*+*o* process provides the primary contribution, while the *o*+*a*→*o*+*a* process yields a secondary but non-negligible contribution. In contrast, the direct *o*→*o*+*a*+*a* and *o*→*a*+*a*+*a* decays play a minor role. These effects are particularly pronounced for TO phonons (Fig. S8b and d). This can be attributed to their 2-fold degeneracy, large optical-deformation potentials, and high ph-ph scattering rates (Figs S15 and S16). These factors enable TO phonons to relax efficiently within the lattice once they become highly populated, and therefore, can absorb substantial energy from charge carriers, helping sustain a net energy flow from the electronic subsystem to the lattice through TO-assisted e-ph scattering. More interestingly, we find that BSb follows the same underlying mechanism as BAs, highlighting the general applicability of the concept of hot-phonon delocalization in wide-phonon-gap semiconductors. A key difference is that the TO-assisted e-ph coupling strength in BSb is weaker than in BAs, particularly for the dominant long-wavelength phonons. Therefore, TO phonons play a less dominant role in carrier cooling in BSb, although they remain important.

As discussed above, optical-phonon decay in wide-phonon-gap semiconductors is fundamentally different from the conventional understanding. In BN and BP, the reduction of hot-phonon accumulation is achieved mainly through three-phonon emission processes, including the *o*→*o*+*a* channels (Ridley- and Vallée-Bogani-type) and the *o*→*a*+*a* channels (Klemens-type). By contrast, the *o*→*o*+*o* processes (Barman-Srivastava-type) are forbidden by energy conservation. Although initially less-occupied optical modes can still become populated in momentum space, this delocalization necessarily accompanies a direct transfer of energy from optical to acoustic phonons, namely, energy redistribution across the phonon subsystem. This is evidenced in Figs S4b and S5b, where the branch-resolved phonon temperatures are close to the lattice temperature, indicating strong mutual relaxation between optical and acoustic phonons and, consequently, no large temperature difference. In BAs and BSb, the above three-phonon scattering channels are largely suppressed due to the presence of the large *a*-*o* gaps. In this regime, four-phonon redistribution processes become dominant, primarily *o*+*o*→*o*+*o* and *o*+*a*→*o*+*a* processes. For the latter, there is a small but non-negligible probability that the scattered optical phonon gains energy; thus, a reduction of optical-phonon energy is not guaranteed in every event. Moreover, our simulations show that the emission channels *o*→*o*+*a*+*a* and *o*→*a*+*a*+*a* have negligible impact on thermalization. Therefore, the four-phonon-induced redistribution of optical phonons mainly reflects transfer among optical modes in momentum space, with a weaker connection to direct energy loss. Here, the term ‘delocalization’ refers to the redistribution of optical phonons in momentum space rather than energy relaxation. Consistent with this interpretation, Figs S6b and S7b, as well as the longer-time results in Fig. S24b and d show that optical and acoustic phonons relax more independently in BAs and BSb, resulting in a pronounced separation between their branch-resolved temperatures.

The importance of four-phonon processes has been theoretically established, with their impact on charge-carrier temperature positively correlated with the magnitude of the ph-ph matrix elements, as reflected in the phonon scattering rates (or equivalently, phonon lifetimes). To validate this effect, femtosecond stimulated Raman spectroscopy (FSRS) measurements were combined with *ab initio* simulations to determine LO phonon lifetimes in a *p*-type BAs sample (see Methods for crystal characterization and experiment details). The primary purpose of the FSRS measurements is to verify that our theoretical model does not overestimate the four-phonon effect by confirming that the *ab initio* calculations yield realistic phonon lifetimes limited by ph-ph interactions. Moreover, because hot phonons predominantly accumulate near the Brillouin zone center, where atoms vibrate out of phase, FSRS can directly probe the lifetimes of these zone-center optical phonons. In the experiment, hot electrons and phonons were excited using high-energy photons at a wavelength of 400 nm [61]. The hot-phonon dynamics were monitored using a pair of beams at 795 nm (Raman pump) and 753 nm (probe), whose energy difference matches the LO/TO phonon modes [62]. To remove the baseline signal associated with the dual excitation (400 nm and 795 nm), non-resonant measurements at 746 nm and 762 nm were performed and subtracted [63,64]. The hot-phonon dynamics at 300 K and 400 K are presented in Fig. 2c. Phonon relaxation was fitted using a fixed instrument response function (IRF) determined from the transient transmission signal of a sapphire substrate placed at the sample position. The extracted Gaussian IRF width is *σ* = 0.198 ps. With this fixed IRF, we obtained LO-phonon lifetimes of 20.1 ± 1.3 ps at 300 K, 14.1 ± 1.1 ps at 350 K, and 11.4 ± 1.0 ps at 400 K. Theoretical predictions show that four-phonon scattering reduces the phonon lifetime by nearly two orders of magnitude at room temperature (from 3998.8 ps to 27.4 ps; Fig. 2d). After accounting for natural isotope abundance, the predicted lifetimes are 16.7 ps at 300 K, 12.8 ps at 350 K, and 10.1 ps at 400 K, in good agreement with the FSRS measurements.

Previous studies have shown that isotope disorder can decrease phonon lifetimes and thermal conductivity in BAs [50,52]. The calculated phonon-isotope scattering rates for most optical modes are on the order of ∼1–10 THz, corresponding to characteristic timescales of ∼0.1–1 ps, suggesting that isotope scattering can begin to affect optical-phonon relaxation on picosecond timescales. Physically, isotope disorder introduces additional elastic scattering channels for phonons, which can facilitate the redistribution of hot-phonon populations in momentum space and potentially accelerate the delocalization of hot phonons. A quantitative assessment, however, would require further work by explicitly incorporating isotope scattering into the phonon BTE and tracking its impact on the time-dependent phonon occupations. Overall, these results of dominant decay channels identify higher-order lattice anharmonicity as a key factor governing carrier thermalization in BAs and BSb.

### Phonon relaxation dynamics with four-phonon couplings

**Figure 3.**
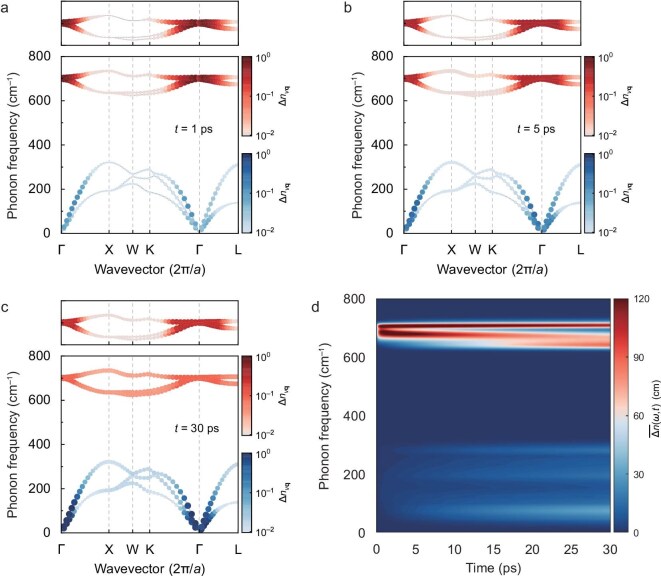
Phonon population augmentation in *p*-type BAs. (a–c) Mode-resolved augmentation of the phonon population at *t* = 1, 5, and 30 ps. Top panels show results excluding four-phonon couplings. Red and blue indicate optical and acoustic phonons, respectively. (d) Time evolution of the averaged phonon population augmentation as a function of phonon frequency.

The mode-resolved phonon populations in BAs provide a direct and quantitative probe of the thermalization dynamics described above. Figure 3a–c shows the augmentation of the phonon occupation number, defined as ${\mathrm{\Delta }}{n}_{\nu {{\bf q}}}( t ) = {n}_{\nu {{\bf q}}}( t ) - {n}_{\nu {{\bf q}}}( {\mathrm{0}} )$, where both the color and size of the symbols represent the buildup of phonon population (see Figs S9–S12 for other boron-group V semiconductors). During the early stage (*t* ≤ 1 ps), the increase in phonon population is only weakly affected by four-phonon decays, because energy exchange is dominated by e-ph coupling. In polar semiconductors, charge carriers predominantly couple to LO phonons through the Fröhlich interaction. Although TO phonons lack the 1/*q* divergence, they also become highly populated, as indicated by the dark red symbols, for the reasons discussed earlier. As the system evolves, the hot phonons accumulated near the Brillouin zone center begin to relax within the lattice subsystem. After 1 ps, their energy is further redistributed to other optical modes through four-phonon couplings. A comparison with the upper panel, which includes only three-phonon interactions, shows that this relaxation is not driven by conventional Barman-Srivastava-type decays, but instead by four-phonon redistribution processes that generate short- to mid-wavelength optical phonons through the annihilation of highly non-thermal phonons. To visualize the redistribution of hot phonons in the frequency domain, we examine the frequency-resolved averaged augmentation of phonon occupation, $\overline {{\mathrm{\Delta }}n} ( {\omega ,\ t} ) = \mathop \sum \nolimits_{\nu {\mathrm{, }}{{\bf q}}} {\mathrm{\Delta }}{n}_{\nu {{\bf q}}}( t )\delta ( {\omega - {\omega }_{\nu {{\bf q}}}} )$, as shown in Fig. 3d (see Figs S17–S19 for other boron-group V semiconductors). The results show that depletion of hot phonons near the zone center is compensated by population buildup over a broader frequency range as thermalization proceeds, indicating efficient spectral redistribution. Neglecting four-phonon processes therefore underestimates the effectiveness of internal thermal relaxation within the optical branches of BAs and BSb, particularly for TO phonons, whose delocalization is not adequately captured by three-phonon decays alone.

For completeness, we also discuss the effects of four-phonon coupling on the relaxation of acoustic modes in *p*-type BAs. As shown in Fig. S25a, four-phonon scattering rates become comparable to three-phonon rates in the 150–200 cm^−1^ frequency range, with the dominant contributions coming from the *a*+*a*→*a*+*a* and *a*+*o*→*a*+*o* channels (red and blue symbols), both of which are redistribution-type processes. Unlike optical phonons, whose population increase is primarily driven by e-ph emission, the generation of acoustic phonons mainly comes from the decay of optical phonons. To isolate the net effect of four-phonon-induced redistribution, Fig. S25b shows the mode-resolved net change in phonon occupation induced by four-phonon coupling at different times. It is found that the occupation numbers in the 150–200 cm^−1^ frequency range decrease when four-phonon scattering is included, indicating that four-phonon coupling efficiently transfers population out of these acoustic modes into other states through the *a*+*a*→*a*+*a* and *a*+*o*→*a*+*o* redistribution channels.

**Figure 4.**
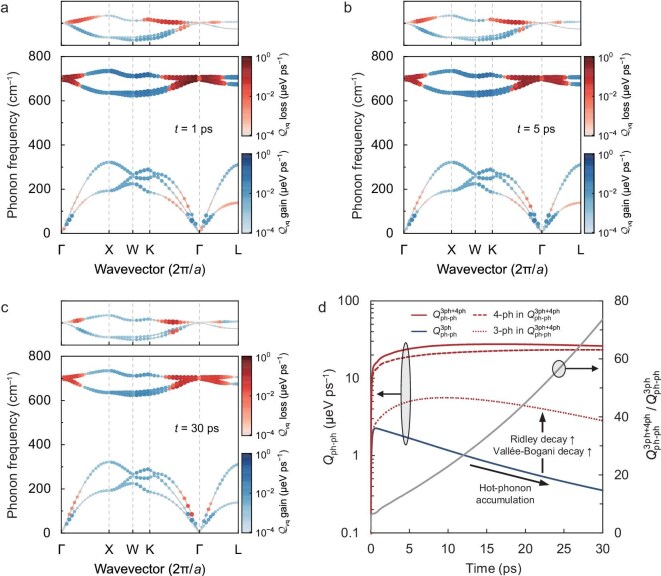
Energy exchange rate within the lattice subsystem of *p*-type BAs. (a–c) Mode-resolved phonon energy exchange rates due to ph-ph interactions at *t* = 1, 5, and 30 ps. Red and blue indicate phonon energy loss and gain, respectively. (d) Time evolution of the total energy exchange rate of optical phonons due to ph-ph interactions. Solid red and blue lines represent results with and without four-phonon couplings, respectively. Dotted and dashed lines denote contributions from three- and four-phonon processes. The gray line shows the ratio of energy exchange rates with versus without four-phonon couplings.

To assess energy flow through specific channels, we analyze the mode-resolved ph-ph energy exchange rate, ${\mathcal{Q}}_{\nu {{\bf q}}}( t ) = N_{{\bf q}}^{\ - {\mathrm{1}}}\hbar {\omega }_{\nu {{\bf q}}}\partial {n}_{\nu {{\bf q}}}/\partial t{|}_{{\mathrm{ph \hbox{-} ph}}}$, as shown in Fig. 4a–c (see Figs S20–S23 for other boron-group V semiconductors). In these plots, red and blue denote net energy loss and gain, respectively. As illustrated in the upper panels, the three-phonon Klemens and Barman-Srivastava decays at the zone center are forbidden in BAs by energy conservation. A hot LO phonon can instead relax through Ridley- or Vallée-Bogani-type processes, converting into a TO or another LO phonon while emitting an acoustic phonon with a frequency below 100 cm^−1^. The TO phonons exhibit poor relaxation under three-phonon scattering. This behavior can be attributed to the complete prohibition of *o*→*o*+*o* processes and to the highly asymmetric *o*→*o*+*a* and *o*+*a*→*o* processes, which favor acoustic-phonon absorption over emission by several orders of magnitude (see gray triangles in Fig. 2a). However, this apparent thermalization behavior warrants further examination, as neglecting four-phonon coupling leads to incorrect linewidths. Our simulations reveal that the *o*+*o*→*o*+*o* and *o*+*a*→*o*+*a* scatterings fundamentally alter the energy exchange dynamics of these non-thermal phonons. First, phonon decay processes at the zone center become allowed through *o*→*a*+*a*+*a* scattering. Second, long-wavelength LO phonons gain additional channels to transfer energy to other states. Most notably, the non-thermal TO phonons reverse their role from energy acceptors to energy donors during thermalization. These phenomena are also observed in BSb (Fig. S23). The four-phonon-mediated mechanisms enhance intralattice energy exchange on timescales of tens of picoseconds. The total energy loss rate of optical phonons, ${Q}_{{\mathrm{ph \hbox{-} ph}}}( t ) = \mathop \sum \nolimits_{\nu {\mathrm{,\ }}{{\bf q}}} {\mathcal{Q}}_{\nu {{\bf q}}}( t )$, where *ν* denotes the three optical branches, is shown in Fig. 4d [see panel (c) in Figs S4–S7 for other boron-group V semiconductors]. The simulations indicate that four-phonon coupling accelerates optical-phonon energy dissipation by more than 70-fold compared with the case including only three-phonon coupling (from 0.36 to 26.12 *μ*eV/ps). This enhancement is expected to persist, as indicated by the gray line. Furthermore, four-phonon scattering reinforces Ridley- and Vallée-Bogani-type decays by enabling them to occur over a broader range of phonon states, thereby preventing the suppression of three-phonon energy exchange caused by unrealistic hot-phonon accumulation, consistent with the trends observed in phonon occupation augmentation.

### Electron-phonon thermalization with four-phonon couplings

**Figure 5.**
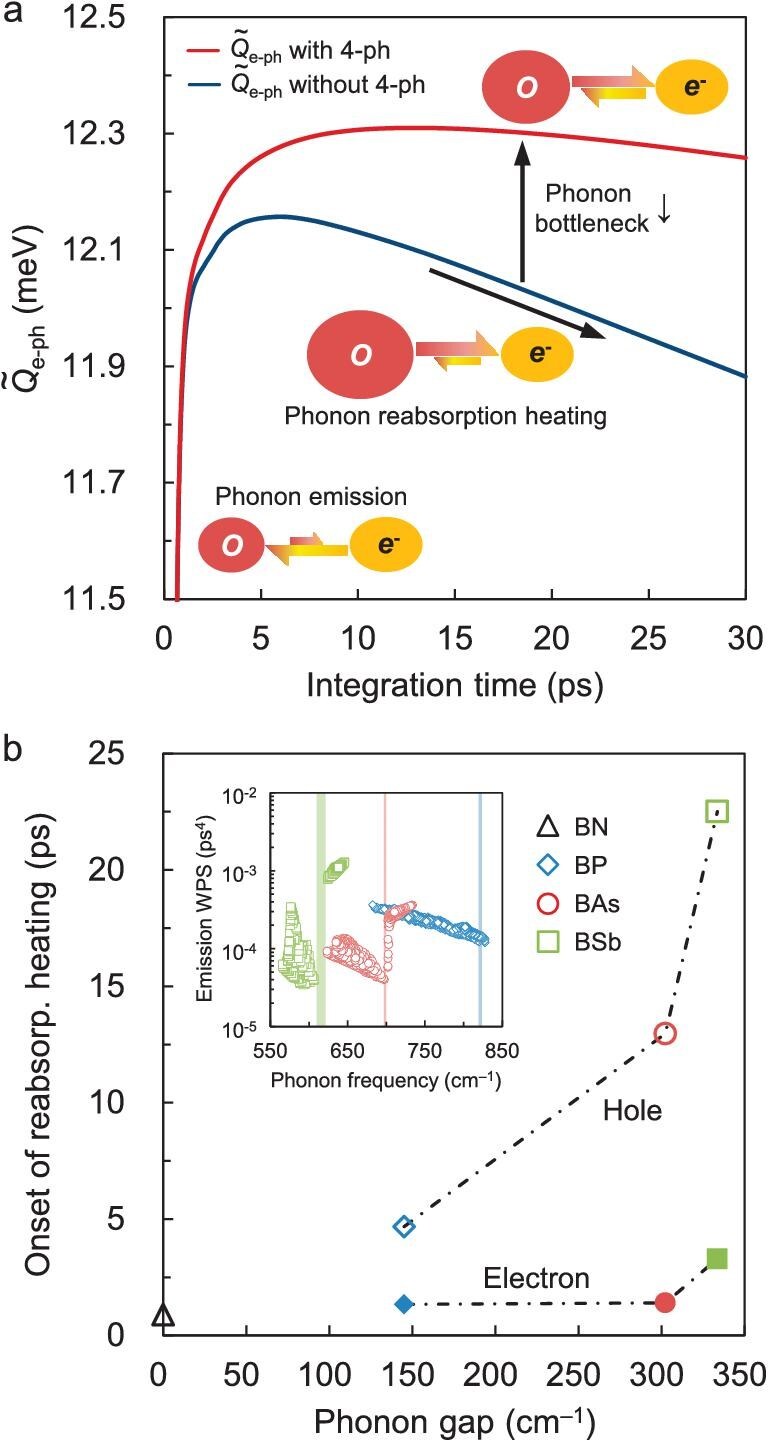
Thermalization dynamics between electronic and lattice subsystems. (a) Cumulative energy transfer from electrons to optical phonons through e-ph interactions in *p*-type BAs. Red and blue lines represent results with and without four-phonon couplings, respectively. Colored arrows schematically illustrate the direction of energy exchange between the electronic and optical-phonon subsystems. The horizontal axis denotes the integration time window starting from *t* = 0. (b) Onset time of phonon reabsorption heating as a function of *a*-*o* gap for the four boron-group V semiconductors. Open and filled symbols denote hole and electron conduction, respectively. Note that phonon reabsorption heating is a transient effect in *p*-type BN and is absent in *n*-type BN within the simulation window, owing to the lack of a phonon gap. The inset shows the WPS of emission processes of optical modes in BP, BAs, and BSb. Shaded regions indicate LO-TO splitting near the Brillouin zone center.

The mechanism described above highlights an enhancement in energy transfer from electrons to the lattice, which can be quantitatively evaluated by examining the cumulative energy transferred to the optical phonon branches, defined as ${\mathrm{\ }}{\tilde{Q}}_{{\mathrm{e \hbox{-} ph}}}( \tau ) = \mathop \smallint \nolimits_{\mathrm{0}}^\tau {Q}_{{\mathrm{e \hbox{-} ph}}}( t )dt$. Figure 5a presents the results for BAs (see panel (d) in Figs S4–S7 for other boron-group V semiconductors). In the early stage of thermalization, energy transfer is dominated by phonon emission processes, leading to the generation of a large population of hot phonons. During this period, the cumulative energy transfer remains nearly identical regardless of whether four-phonon scattering is included. At ∼1 ps, the two curves begin to diverge, corresponding to the onset of pronounced internal thermal relaxation within the lattice. At this stage, the slopes of both curves decrease, as phonon absorption becomes increasingly significant and begins to partially offset the net energy flow. As the system continues to evolve, the cumulative energy transfer reaches a maximum. Beyond this point, the slope becomes negative, indicating a reversal in energy flow, i.e. energy is transferred from hot phonons back to the electronic subsystem, which is a hallmark of the phonon bottleneck effect. Simulations show that, when evaluated at a characteristic time of 1 ps, the inclusion of four-phonon coupling increases the peak energy transfer by more than 55% (from 0.25 to 0.39 meV). Furthermore, the onset of phonon reabsorption heating is delayed from 6.0 to 12.9 ps (in BSb, from 10.3 to 22.6 ps). Although four-phonon scattering does not eliminate reabsorption heating in BAs, the cumulative energy transfer exhibits only a slight decline from its peak value. These results provide direct evidence that higher-order phonon decay channels can effectively mitigate phonon bottleneck effects by sustaining forward energy flow over extended timescales.

The mode-resolved net change in accumulated e-ph energy transfer induced by four-phonon couplings at different times provides further evidence (Figs S26–S29). In these plots, an ‘increase’ or a ‘decrease’ for a given mode indicates whether the accumulated energy transferred with four-phonon processes included is larger or smaller than that obtained when only three-phonon processes are considered. The results show that the long-wavelength optical phonons near the Γ point receive more energy from the electronic subsystem when four-phonon coupling is included, whereas some mid-wavelength optical phonons exhibit the opposite trend. This behavior occurs because four-phonon scattering redistributes the accumulated non-equilibrium long-wavelength optical phonons into less-occupied optical modes, that is, hot-phonon delocalization. For acoustic phonons, the net energy transferred from electrons decreases when including four-phonon scattering. This is because the additional channels accelerate lattice thermal relaxation by promoting the decay rate of high-frequency phonons into lower-frequency ones, which in turn reduces the phase space for electron-acoustic-phonon scattering.

While the thermalization dynamics depend on multiple intertwined factors, including electron energy, phonon frequency, and coupling parameters for e-ph and ph-ph interactions, the dominance of four-phonon coupling in semiconductors ultimately stems from a pronounced *a*-*o* gap, which suppresses the available scattering phase space. We observed that the onset time of phonon reabsorption heating correlates positively with the *a*-*o* gap, as shown in Fig. 5b. This trend can be understood as follows. An *a*-*o* gap suppresses the phase space of ph-ph emission channels, making optical-phonon decay progressively more difficult as the gap increases. The weighted phase space (WPS) [65] for ph-ph emission processes of optical modes is shown in the inset of Fig. 5b. It can be seen that the WPS generally decreases with increasing *a*-*o* gap, particularly near the Brillouin-zone center, where non-equilibrium hot phonons predominantly populate. Consequently, when the WPS is more strongly suppressed, optical modes retain higher effective temperatures or populations for a longer time (Figs S6b and S7b). This, in turn, slows charge-carrier cooling (Figs S6a and S7a) and prolongs the competition between e-ph emission and absorption, delaying the transition to a net energy flow from optical phonon to electron.

To provide a more quantitative explanation, we fit the hole temperatures using a bi-exponential function, ${T}_{\mathrm{e}}( t ) = {A}_{\mathrm{0}}{\mathrm{ + }}{A}_{\mathrm{1}}{{\mathrm{e}}}^{ - t/{\tau }_{\mathrm{1}}}{\mathrm{ + }}{A}_{\mathrm{2}}{{\mathrm{e}}}^{ - t/{\tau }_{\mathrm{2}}}$, where ${\tau }_{\mathrm{1}}$ and ${\tau }_{\mathrm{2}}$ characterize the timescales of e-ph interaction and hot-phonon relaxation, respectively [66]. The fitted parameters are summarized in Table S1. Both decay constants consistently increase with the *a*-*o* gap, indicating that a larger gap leads to slower charge-carrier cooling and slower hot-phonon relaxation. Therefore, when treating the optical branch as a whole, a longer time is required for ${T}_{\mathrm{e}}$ to approach the effective temperature of the optical phonons, and the onset of reabsorption heating is correspondingly delayed, consistent with the trend in Fig. 5b. These results further highlight that explicitly including four-phonon coupling is essential for accurately predicting the occurrence of phonon reabsorption heating in wide-phonon-gap semiconductors.

## CONCLUSIONS

In summary, this study demonstrated that four-phonon processes should be rigorously considered in modeling carrier thermalization in semiconductors with significant phonon gaps. Their effect on thermalization dynamics is to redistribute momentum from non-thermal phonons to other less-occupied optical modes, thereby reducing the accumulation of hot phonons. This delocalization effect simultaneously enhances energy transfer mediated by three-phonon decays and delays the onset of phonon reabsorption heating. High-energy charge carriers can thus dump more energy into the lattice, mitigating the phonon bottleneck. These non-equilibrium phenomena in wide-phonon-gap semiconductors cannot be sufficiently described within a three-phonon-only framework, which may overestimate hot-phonon accumulation and underestimate charge-carrier cooling efficiency. Our results highlight the importance of higher-order anharmonic decays in understanding multi-carrier thermalization. It is also worthwhile to explore the influence of four-phonon coupling on mode-resolved electron thermalization beyond the conventional assumption of Fermi-Dirac statistics for the electronic subsystem.

## METHODS

### *Ab initio* calculations

The simulations employed fully relativistic optimized norm-conserving Vanderbilt pseudopotentials, with the Perdew-Burke-Ernzerhof parameterization of the exchange-correlation functional [67–69]. Spin-orbit coupling was included, as it is essential for accurately describing *p*-type conduction due to the presence of the split-off band in zinc-blende crystals. All calculations were performed using the Quantum ESPRESSO package [70] and an in-house developed code integrating ShengBTE [71,72] and EPW [73]. Parts of the implementation were informed by the Perturbo code [74]. See Note S2 for the details of the computational parameters and the validation against the experimental reports in ref. [59].

### Sample synthesis and characterization

The BAs sample was synthesized using the chemical vapor transport method described in ref. [48]. Spontaneous Raman scattering was used to characterize the impurity and isotope levels of the samples. See Note S4 for the characterization details.

### Femtosecond stimulated Raman scattering measurement

FSRS was employed to investigate the lifetime of hot phonons in BAs at temperatures of 300 K, 350 K, and 400 K. A schematic of the experimental setup is shown in Fig. S2. Briefly, a femtosecond 400 nm laser beam was used to excite hot carriers in BAs. A supercontinuum white light source (450–780 nm) and a narrowband beam at 795 nm (FWHM ∼3 nm) served as the probe and Raman pump beams, respectively. A mechanical chopper modulated the phonon pump beam to extract the Raman loss, calculated as $( {{I}_{{pump} - on} - {I}_{{pump} - of\!f}} )/{I}_{{pump} - of\!f}$. A stepper motor controlled the time delay between the carrier pump and the other two beams. Transient absorption dynamics at 753 nm (corresponding to 700 cm^–1^), which contain mixed contributions from phonons and carriers (${D}_m$), along with pure carrier dynamics in the ranges of 730–751 nm and 755–780 nm (${D}_c$), were simultaneously recorded. Pure phonon dynamics (${D}_p$) were then extracted using the relation ${D}_p = {D}_m - {D}_c$. Details of experimental procedures are provided in Note S5; FSRS spectra are shown in Fig. S3.

## Supplementary Material

nwag224_Supplemental_Files
