# Supplementary material for: Higher-order lattice anharmonicity reshaping non-equilibrium carrier dynamics in wide-phonon-gap semiconductors
Source: Natl Sci Rev. 2026 Apr 20;13(12):nwag224. doi: 10.1093/nsr/nwag224 (PMC13292162; doi:10.1093/nsr/nwag224)
Supplement: nwag224_Supplemental_Files [file nwag224_supplemental_files.zip › Supplementary Information.pdf]

Supplementary Information

Higher-order lattice anharmonicity reshaping non-equilibrium carrier dynamics in wide-phonon-gap semiconductors

Te-Huan Liu[1,†,*], Xin Qian[1,†], Shuai Yue[2,†,*], Ming Li[1], Zhichun Liu[1], Bai Song[3], Fei Tian[4], Xinfeng Liu[2], and Ronggui Yang[3,*]

[1]*School of Energy and Power Engineering, Huazhong University of Science and Technology, Wuhan, 430074, China*

[2]*Chinese Academy of Sciences Key Laboratory of Standardization and Measurement for Nanotechnology, National Center for Nanoscience and Technology, Beijing 100190, China*

[3]*College of Mechanics and Engineering Science, Peking University, Beijing 100871, China*

[4]*School of Materials Science and Engineering, Sun Yat-sen University, Guangzhou, 510006, China*

[*]Corresponding authors. thliu@hust.edu.cn; yueshuai@nanoctr.cn; ronggui@pku.edu.cn

[†]Equally contributed to this work.

Content

Supplementary Notes 3

Supplementary Figures 12

Supplementary Table 41

Supplementary Movies 42

Supplementary References 44

Note S1. Collision terms for multi-carrier couplings

The time-dependent electron temperature and phonon occupation numbers were determined by solving the coupled electron and phonon BTEs, given in Eqs. (3) and (4) in the main text. The evolution of the electron temperature is governed exclusively by ph-e interactions, whereas the phonon occupation numbers depend on all phonon-related couplings considered in this work.

To solve the coupled BTEs, the collision terms arising from ph-e, three-phonon, and four-phonon interactions were evaluated explicitly. The variation of the phonon population due to ph-e scattering is given by [S1]:

$$\left.\frac{\partial n_{\nu\mathbf{q}}}{\partial t}\right|_{\text{ph-e}} = \frac{2\pi g_{\text{e}}}{\hbar}\frac{1}{N_{\mathbf{k}}}\sum_{n,n',\mathbf{k}}|g|^2\begin{bmatrix} n_{\nu\mathbf{q}}f_{n\mathbf{k}}\left(1-f_{n'\mathbf{k}'}\right) \\ -\left(1+n_{\nu\mathbf{q}}\right)\left(1-f_{n\mathbf{k}}\right)f_{n'\mathbf{k}'}\end{bmatrix}\delta\left(\hbar\omega_{\nu\mathbf{q}}+\varepsilon_{n\mathbf{k}}-\varepsilon_{n'\mathbf{k}'}\right), \tag{S1}$$

with the matrix elements expressed as:

$$g_{nn'}(\mathbf{k},\mathbf{q}) = \left(\frac{\hbar}{2m_0\omega_{\nu\mathbf{q}}}\right)^{1/2}\left\langle n'\mathbf{k}'\middle|\delta V_{\nu\mathbf{q}}(\mathbf{r})\middle|n\mathbf{k}\right\rangle. \tag{S2}$$

Here, g_{e} denotes the electron degeneracy (equal to 1 if spin-orbit coupling is numerically included, and 2 if it is not), m_0 is a reference mass, and $\delta V_{\nu\mathbf{q}}(\mathbf{r})$ is the phonon-perturbed potential in real space. The variation of the phonon population due to three-phonon scattering is given by [S1]:

$$\left.\frac{\partial n_{\nu\mathbf{q}}}{\partial t}\right|_{\text{3-ph}} = \frac{2\pi}{\hbar}\frac{1}{N_{\mathbf{q}'}}\sum_{\nu',\nu'',\mathbf{q}'}|\mathcal{M}|^2\begin{bmatrix} \left(n_{\nu'\mathbf{q}'}-n_{\nu''\mathbf{q}''}\right) \\ \times\,\delta\left(\hbar\omega_{\nu\mathbf{q}}+\hbar\omega_{\nu'\mathbf{q}'}-\hbar\omega_{\nu''\mathbf{q}''}\right) \\ +\frac{1}{2}\left(n_{\nu'\mathbf{q}'}+n_{\nu''\mathbf{q}''}+1\right) \\ \times\,\delta\left(\hbar\omega_{\nu\mathbf{q}}-\hbar\omega_{\nu'\mathbf{q}'}-\hbar\omega_{\nu''\mathbf{q}''}\right)\end{bmatrix}, \tag{S3}$$

with the matrix elements defined as:

$$\mathcal{M}_{\nu\nu'\nu''}(\mathbf{q},\mathbf{q}') = \left(\frac{\hbar}{2}\right)^{3/2} \sum_{i,j,k} \sum_{\alpha,\beta,\gamma} \Phi_{ijk}^{\alpha\beta\gamma} \frac{\xi_{i,\nu\mathbf{q}}^{\alpha} \xi_{j,\nu'\mathbf{q}'}^{\beta} \xi_{k,\nu''\mathbf{q}''}^{\gamma}}{\sqrt{M_i M_j M_k \omega_{\nu\mathbf{q}} \omega_{\nu'\mathbf{q}'} \omega_{\nu''\mathbf{q}''}}} e^{i\mathbf{q}'\cdot\mathbf{r}_j} e^{i\mathbf{q}''\cdot\mathbf{r}_k}, \quad \text{(S4)}$$

where $\mathbf{\Phi}$ denotes the third-order force constants, ξ the phonon eigenvectors, $\mathbf{r}$ the atomic positions, and M the atomic masses. Indices i, j, k refer to Cartesian coordinates, and α, β, γ label basis atoms. The variation of phonon population due to four-phonon scattering is given by [S1]:

$$\left.\frac{\partial n_{\nu\mathbf{q}}}{\partial t}\right|_{\text{4-ph}} = \frac{2\pi}{\hbar} \frac{1}{N_{\mathbf{q}'} N_{\mathbf{q}''}} \sum_{\nu',\nu'',\nu''',\mathbf{q}',\mathbf{q}''} |\mathcal{N}|^2 \begin{bmatrix} \frac{1}{2}(1+n_{\nu'\mathbf{q}'})(1+n_{\nu''\mathbf{q}''}) n_{\nu'''\mathbf{q}'''} \\ \times\, \delta(\hbar\omega_{\nu\mathbf{q}} + \hbar\omega_{\nu'\mathbf{q}'} + \hbar\omega_{\nu''\mathbf{q}''} - \hbar\omega_{\nu'''\mathbf{q}'''}) \\ + \frac{1}{2}(1+n_{\nu'\mathbf{q}'}) n_{\nu''\mathbf{q}''} n_{\nu'''\mathbf{q}'''} \\ \times\, \delta(\hbar\omega_{\nu\mathbf{q}} + \hbar\omega_{\nu'\mathbf{q}'} - \hbar\omega_{\nu''\mathbf{q}''} - \hbar\omega_{\nu'''\mathbf{q}'''}) \\ + \frac{1}{6} n_{\nu'\mathbf{q}'} n_{\nu''\mathbf{q}''} n_{\nu'''\mathbf{q}'''} \\ \times\, \delta(\hbar\omega_{\nu\mathbf{q}} - \hbar\omega_{\nu'\mathbf{q}'} - \hbar\omega_{\nu''\mathbf{q}''} - \hbar\omega_{\nu'''\mathbf{q}'''}) \end{bmatrix}, \quad \text{(S5)}$$

with the matrix elements given by:

$$\mathcal{N}_{\nu\nu'\nu''\nu'''}(\mathbf{q},\mathbf{q}',\mathbf{q}'') = \left(\frac{\hbar}{2}\right)^{2} \sum_{i,j,k,l} \sum_{\alpha,\beta,\gamma,\theta} \Psi_{ijkl}^{\alpha\beta\gamma\theta} \frac{\xi_{i,\nu\mathbf{q}}^{\alpha} \xi_{j,\nu'\mathbf{q}'}^{\beta} \xi_{k,\nu''\mathbf{q}''}^{\gamma} \xi_{l,\nu'''\mathbf{q}'''}^{\theta}}{\sqrt{M_i M_j M_k M_k \omega_{\nu\mathbf{q}} \omega_{\nu'\mathbf{q}'} \omega_{\nu''\mathbf{q}''} \omega_{\nu''\mathbf{q}''}}}$$
$$\times\, e^{i\mathbf{q}'\cdot\mathbf{r}_j} e^{i\mathbf{q}''\cdot\mathbf{r}_k} e^{i\mathbf{q}'''\cdot\mathbf{r}_l}, \quad \text{(S6)}$$

where $\mathbf{\Psi}$ denotes the fourth-order force constants.

Equations (S1)–(S6) were obtained from first-principles calculations. The electron and phonon eigenvalues and eigenvectors were feld fixed throughout the simulations, whereas the phonon occupation numbers were updated at each time step, rendering the joint density of states time-dependent. The updated collision terms for ph-e, three-phonon, and four-phonon interactions were then used to compute the electron temperature and phonon distributions at the subsequent time step, enabling the time evolution of electron and phonon thermalization.

Note S2. Parameters and validation of *ab initio* calculations

We outline the computational settings used to calculate the electron and phonon properties. For electronic structure calculations, a uniform 16 × 16 × 16 **k**-mesh and a plane-wave cutoff energy of 100 Ry were used for self-consistent field calculations, with a total energy convergence threshold of 10^{-12} Ry. Phonon frequencies, eigenvectors, and phonon-perturbed potentials were computed using density functional perturbation theory on an 8 × 8 × 8 **q**-mesh, with a stricter energy convergence threshold of 10^{-20} Ry. Third-order force constants were obtained from atomic forces calculated using a 4 × 4 × 4 supercell combined with a 3 × 3 × 3 **k**-mesh, with interactions truncated beyond the sixth-nearest neighbors. Fourth-order force constants were computed using the same settings, except that the interaction range was truncated at the third-nearest neighbors.

All collision integrals were computed on a dense 36 × 36 × 36 **q**-mesh. For the ph-e interactions, each phonon mode interacted with electrons sampled on a dense 108 × 108 × 108 **k**-mesh. Electron Hamiltonians, dynamical matrices, and carrier coupling matrix elements on these dense grids were interpolated from coarse-grid *ab initio* data using Wannier-Fourier interpolation. The computed ph-ph and ph-e scattering rates, as well as e-ph matrix elements, are shown in Figs S13–S16. After determining the collision terms, the time-dependent electron temperature and phonon occupation numbers were obtained by solving the coupled BTEs (Eqs. (3) and (4) in the main text). A time step of 1 fs was used to ensure numerical convergence.

To validate our simulations against the experimental results in Ref. [S2], we calculated the time evolution of charge-carrier and phonon temperatures for BAs over a 100 ps window (Fig. S24), which is also a timescale relevant to the effective switching time of MOSFETs in advanced logic chips. Even with four-phonon

scattering included, neither *p*-type nor *n*-type BAs reaches global thermal equilibrium within 100 ps (*p*-type: T_e = 374 K, T_{ph} = 343 K; *n*-type: T_e = 391 K, T_{ph} = 360 K), as the cooling curves flatten with a temperature difference of ~30 K. This result supports the view that a fraction of electrons or holes can remain in high-energy states. Moreover, lattice thermal relaxation remains incomplete, as evidenced by the significant temperature separation between the optical and acoustic branches. These extended-time simulations indicate that, within the coupled BTE framework, electron and hole temperatures in BAs can remain appreciably higher than the average lattice temperature on the hundred-picosecond timescale, in qualitative agreement with the experimentally reported long-lived hot-carrier transport time (~145–250 ps).

Note S3. Phenomenological model for net absorption rate

We start from Eq. (S9) in Ref. [S2], which was derived to analyze the effect of non-equilibrium phonon populations on the phonon bottleneck. It is expressed as:

$$\Delta\mathcal{P}_{k+q\to k'} = \frac{2\pi}{\hbar}\left|g_{k,q}\right|^2\left(f_k^0 - f_{k'}^0\right)\Delta n_q \delta\left(\varepsilon_k - \varepsilon_{k'} + \hbar\omega_q\right)\delta_{k+q,k'}. \tag{S7}$$

Now we limit the analysis to optical phonons. Assuming that long-wavelength phonons dominate the e-ph interaction, the coupling matrix elements for the Fröhlich and optical-deformation-potential interactions are, respectively, given by [S3]:

$$g_q^{\text{Frö}} = \frac{i}{q}\sqrt{\frac{e^2\hbar\omega_q}{2\Omega}\left(\epsilon_\infty^{-1} - \epsilon_0^{-1}\right)}, \tag{S8}$$

$$g_q^{\text{ODP}} = \sqrt{\frac{\hbar\Xi^2}{2\rho\Omega\omega_q}}. \tag{S9}$$

After being excited by an external field, the electron temperature is elevated during the thermalization process. In this high-temperature limit, the equilibrium electron distribution follows Maxwell-Boltzmann statistics and can be expressed as $f_k^0 = \exp\left(\frac{\varepsilon_F - \varepsilon_k}{k_B T_e}\right)$, where ε_F is the Fermi energy. In a semiconductor with a parabolic band, this becomes: $f_k^0 = \exp\left(\frac{\varepsilon_F - \varepsilon_{V,C}}{k_B T_e} - \frac{\hbar^2 k^2}{2m_e k_B T_e}\right)$, where ε_V and ε_C are the energies at the valence band maximum and conduction band minimum, respectively. Thus, the term of difference of electron distribution in Eq. (S1) becomes:

$$f_k^0 - f_{k'}^0 = e^{\frac{\varepsilon_F - \varepsilon_{V,C}}{k_B T_e}} e^{-\frac{\hbar^2 k^2}{2m_e k_B T_e}}\left(1 - e^{-\frac{\hbar^2 kq\cos\theta}{m_e k_B T_e}} e^{-\frac{\hbar^2 q^2}{2m_e k_B T_e}}\right). \tag{S10}$$

Here, the momentum conservation for e-ph scattering, $k'^2 = k^2 + 2kq\cos\theta + q^2$, is used. It is worth noting that we keep the delta functions in Eq. (S7) because the purpose here is not to integrate the absorption rate over all scattering events to obtain

the total electron scattering rate. In a phenomenological model, which does not rely on any discretized mesh, it can always identify scattering events that satisfy both energy and momentum conservation. Therefore, the delta functions are constraints that characterize when scattering occurs.

Under the assumptions of high electron temperature and the long-wavelength limit for phonons, the last term in Eq. (S10) approaches unity. Substituting Eqs. (S8) to (S10) into Eq. (S7), we obtain the parameterized phonon reabsorption rates:

$$\Delta\mathcal{P}_{k+q\to k'}^{\text{Frö}} = \mathcal{F}\Delta n_q \frac{\pi e^2 \omega_q}{\Omega q^2}\left(\frac{1}{\epsilon_\infty} - \frac{1}{\epsilon_0}\right)\delta\left(\varepsilon_k - \varepsilon_{k'} + \hbar\omega_q\right)\delta_{k+q,k'}, \quad \text{(S11)}$$

$$\Delta\mathcal{P}_{k+q\to k'}^{\text{ODP}} = \mathcal{F}\Delta n_q \frac{\pi \Xi^2}{\rho\Omega\omega_q}\delta\left(\varepsilon_k - \varepsilon_{k'} + \hbar\omega_q\right)\delta_{k+q,k'}, \quad \text{(S12)}$$

where $\mathcal{F} = e^{\frac{\varepsilon_F - \varepsilon_{V,C}}{k_B T_\text{e}}} e^{-\frac{\hbar^2 k^2}{2 m_\text{e} k_B T_\text{e}}}$. Using the mass action law to relate charge concentration to the chemical potential, the exponential prefactor in $\mathcal{F}$ can be rewritten as:

$$\mathcal{F} = n_\text{e}\left(\frac{2\pi\hbar^2}{m_\text{e} k_B T_\text{e}}\right)^{3/2} e^{-\frac{\hbar^2 k^2}{2 m_\text{e} k_B T_\text{e}}}. \quad \text{(S13)}$$

Substituting Eq. (S13) into Eqs. (S11) and (S12) yields Eqs. (5) and (6) in the main text.

Note S4. Characterization of the BAs sample

Before characterization, the sample was cut and polished to remove surface impurities. Small pieces with lateral dimensions of several hundred micrometers and a thickness of approximately 50 μm were obtained (Fig. S1(a)). The measurement spot is indicated by a red dot. Spontaneous Raman spectra were acquired using a confocal micro-Raman system (Institute of Semiconductors, CAS) coupled to a Horiba iHR550 imaging spectrometer and a 20× objective lens (Olympus). A 532 nm single-mode laser was used as the excitation source. To eliminate background signals, reference measurements were performed without the sample to subtract the contributions from the charge-coupled device (CCD) and spectrometer luminescence. As shown in Figure S1(b), a sharp LO/TO phonon peak is observed at 702 cm^{-1}, accompanied by an isotope-related peak at 720 cm^{-1}. The broad Raman features in the range of 1200–1500 cm^{-1} are attributed to second-order Raman scattering. Additionally, the weak scattering signal near 1000 cm^{-1} indicates a low concentration of crystal defects.

Note S5. Procedures of the FSRS experiment

FSRS was employed to investigate phonon dynamics, wherein hot phonons are generated via e-ph coupling following carrier excitation. The experimental setup is illustrated in Fig. S2(a). Briefly, 100 fs, 800 nm laser pulses at 1 kHz (laser system: Astrella, Coherent) were split into three beams. The first beam was spectrally filtered using a bandpass filter to produce a narrowband Raman pump centered at 795 nm, with a full width at half maximum (FWHM) of 2–4 nm. The second beam was focused onto a 3 mm-thick sapphire plate to generate a supercontinuum white light source, which served as the Raman probe. The third beam was frequency-doubled to 400 nm using a BBO crystal and used to excite hot carriers in the sample. All beams were focused onto the sample using a 20× objective lens (Mitutoyo, M Plan Apo SL). The sample was mounted on a temperature-controlled stage (Linkam, LTS 350). A mechanical chopper operating at 500 Hz modulated the Raman pump, and a photodetector array with a spectral resolution of 0.7 nm recorded the probe signal at 1 kHz. The stimulated Raman intensity was defined as $\left(I_{pump\text{-}on} - I_{pump\text{-}off}\right)/I_{pump\text{-}off}$. A motorized delay stage controlled the temporal delay between the carrier excitation pulse and the Raman pump/probe beams, enabling a delay range from –200 ps to 800 ps.

Photon pairs at 753 nm and 795 nm, whose energy difference matches the LO phonon energy, can resonantly interact with lattice vibrations and induce nonlinear absorption at 753 nm when the phonon population is low. Upon phonon excitation via electron-phonon coupling, this absorption is suppressed and gradually recovers as the phonons relax and cool down (Fig. S2(b)).

The FSRS spectra are shown in Fig. S3, revealing both sharp phonon-related features and broader carrier dynamics. Figure S3(a) presents the differential

transmission ($\Delta T/T$) spectra of the probe beam at 300 K. Following hot-carrier excitation, the absorption at 753 nm initially decreases and then recovers over time. Time-resolved spectra at selected delays are shown in Fig. S3(b). At 1 ps, the absorption dip is most weakened, while at later times, it gradually recovers. The transient signal at 753 nm contains contributions from both phonon and carrier dynamics (red arrow), whereas nearby off-resonance wavelengths predominantly reflect carrier dynamics (black arrows). To isolate the phonon-specific component, the average of the signals at nearby off-resonance positions was subtracted from the signal at 753 nm. The resulting pure phonon dynamics are plotted in Fig. 2(b).

FSRS spectra measured at different temperatures are shown in Fig. S3. We measured the instrument response function (IRF) of the FSRS setup by placing a sapphire substrate at the sample position. The extracted IRF width is $\sigma = 0.198$ ps. All phonon dynamics were then fitted using this fixed IRF, which ensures improved consistency and accuracy in modeling the phonon dynamics across all temperatures. The lifetimes are 20.1 ± 1.3 ps at 300 K, 14.1 ± 1.1 ps at 350 K, and 11.4 ± 1.0 ps at 400 K.

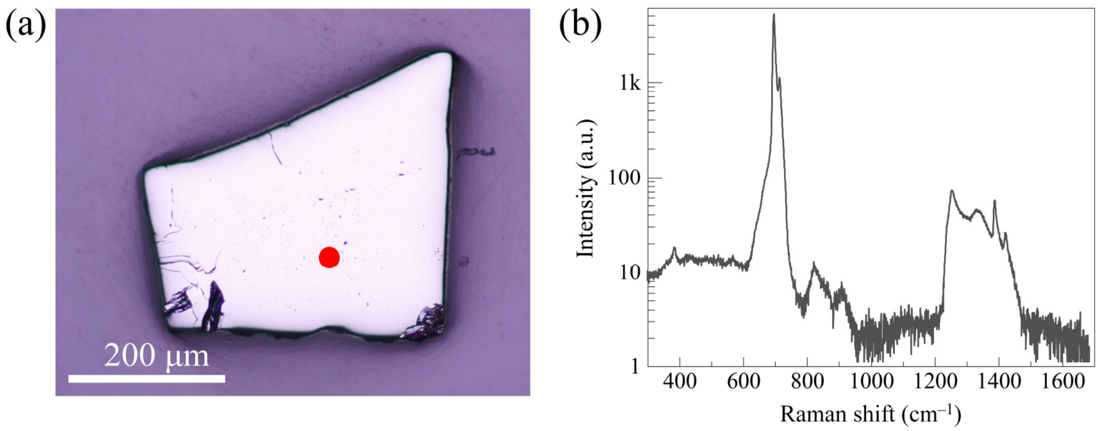

Figure S1. Characterization of the prepared BAs crystal. (a) Optical image of the BAs sample, and (b) the corresponding spontaneous Raman spectrum.

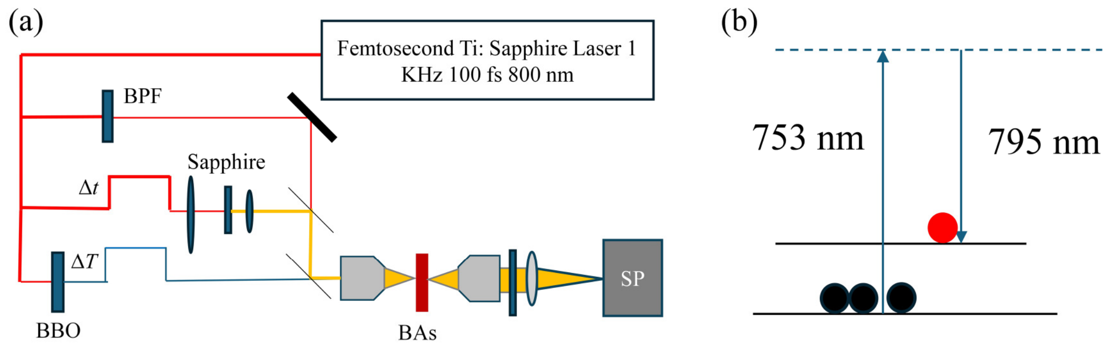

Figure S2. Schematic of the FSRS technique. (a) Experimental setup of the FSRS system. (b) Illustration of the mechanism for hot phonon detection.

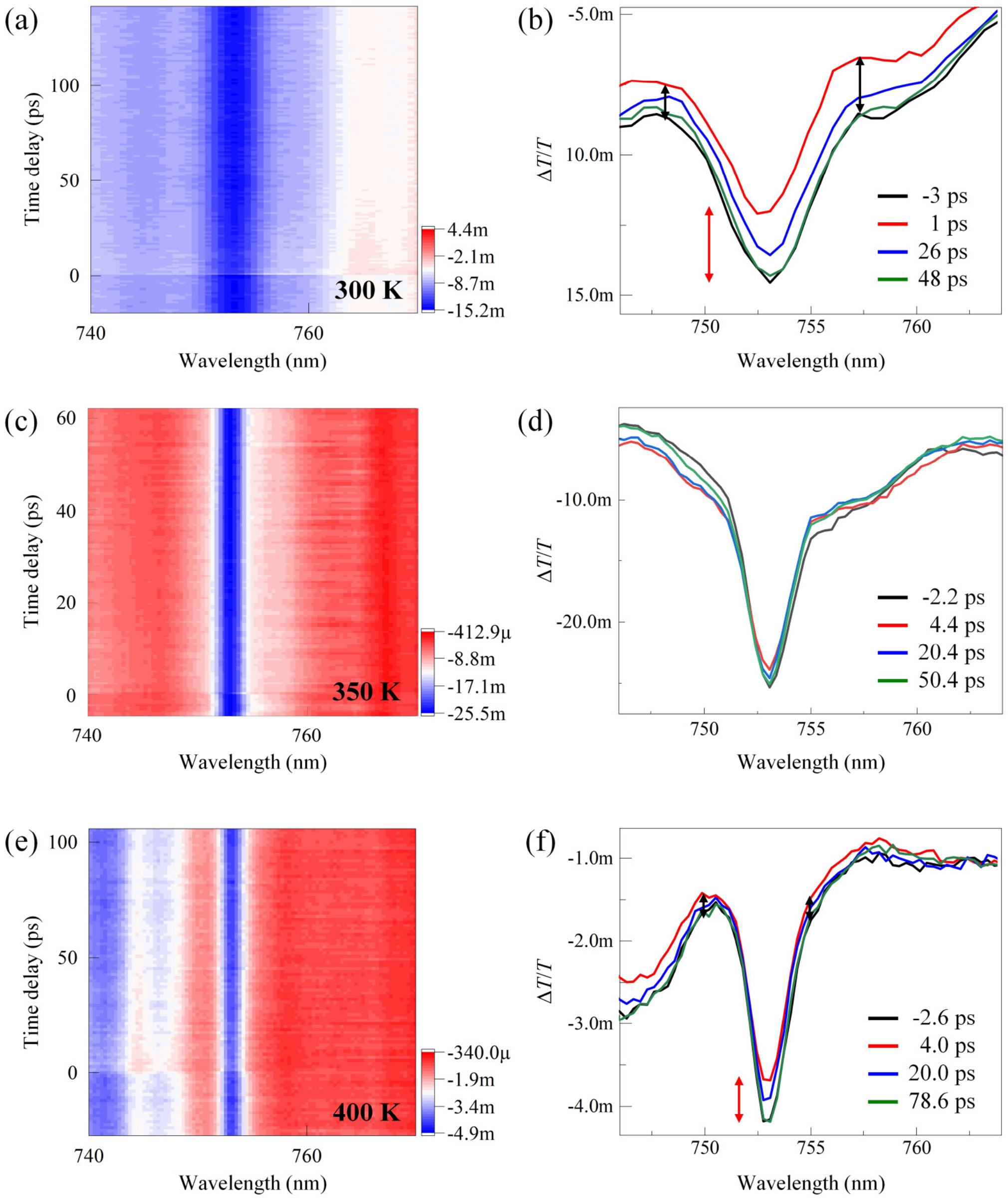

Figure S3. FSRS spectra of BAs at 300, 350, and 400 K. (a) FSRS spectra of BAs at 300 K. (b) Spectra at different time delays. The red arrow indicates a combined signal containing both phonon and carrier contributions, while the black arrows indicate signals primarily associated with carriers. (c) FSRS spectra of BAs at 350 K. (d) Spectra at different time delays at 350 K. (e) FSRS spectra of BAs at 400 K. (f) Spectra at different time delays at 400 K.

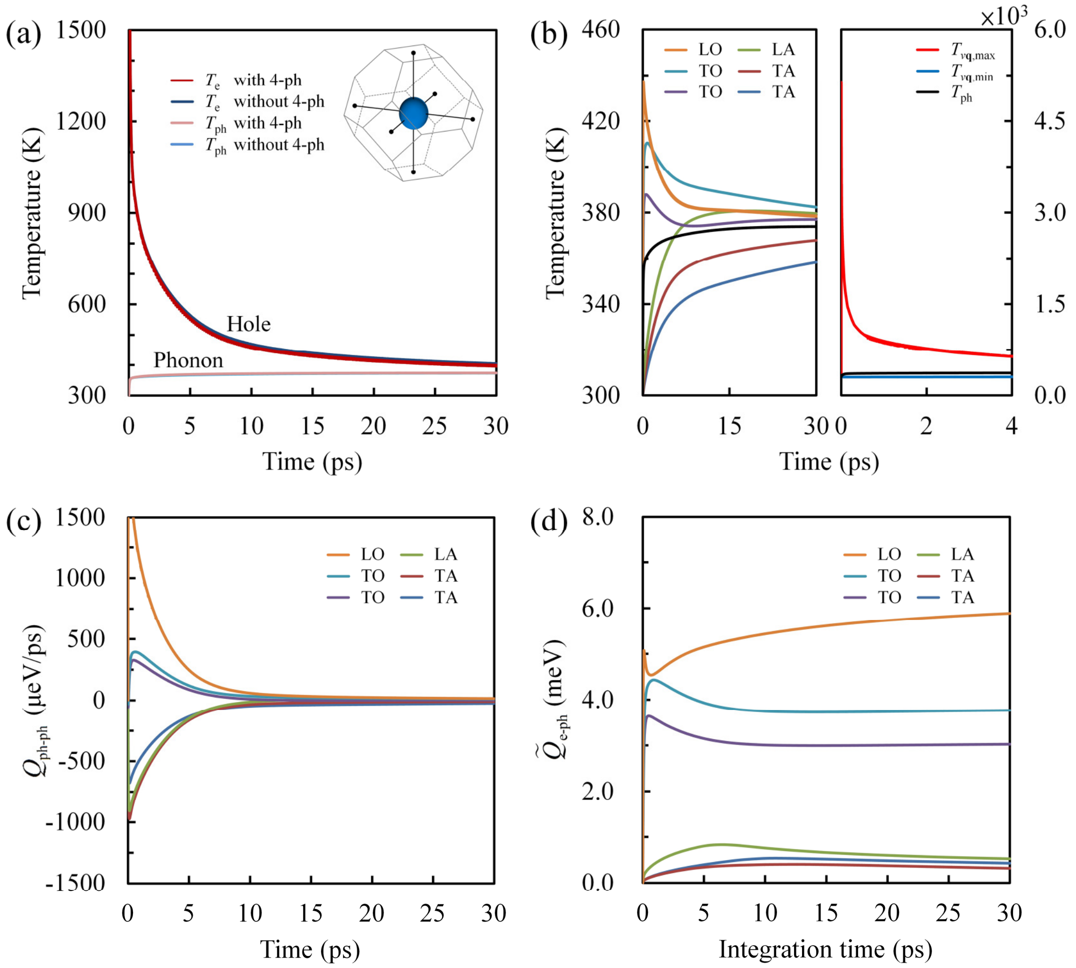

Figure S4. Carrier temperatures and energy transfer of BN under the *p*-type condition. (a) Time evolution of the temperatures of holes and phonons. (b) Time evolution of the branch-resolved phonon temperatures (left panel); the maximum and minimum temperatures among each phonon mode at each time step (right panel). (c) Time evolution of the branch-resolved phonon energy exchange rate due to ph-ph interactions. Positive values represent energy loss (transferring energy to other branches), and negative values represent energy gain. (d) Time evolution of the branch-resolved accumulation of energy transfer from electrons to lattice due to ph-e interactions.

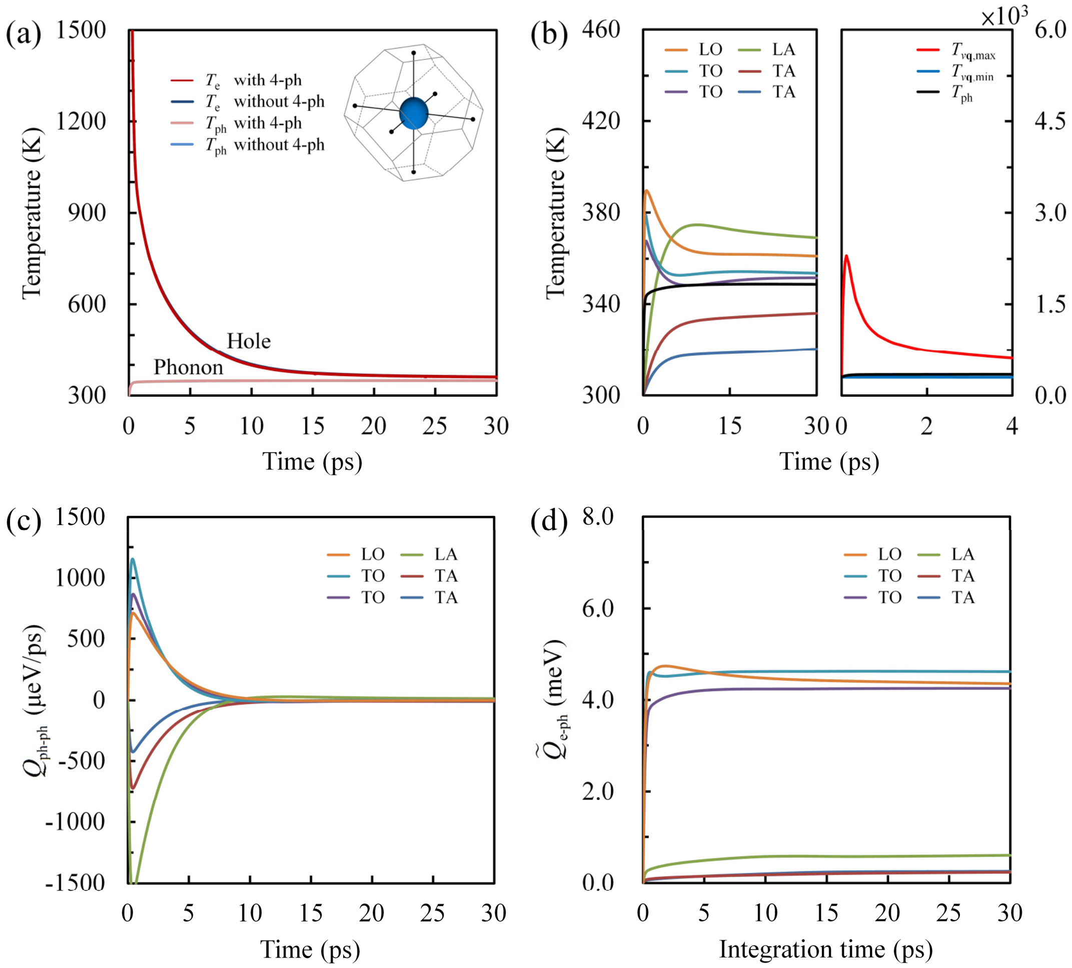

Figure S5. Carrier temperatures and energy transfer of BP under the *p*-type condition. (a) Time evolution of the temperatures of holes and phonons. (b) Time evolution of the branch-resolved phonon temperatures (left panel); the maximum and minimum temperatures among each phonon mode at each time step (right panel). (c) Time evolution of the branch-resolved phonon energy exchange rate due to ph-ph interactions. Positive values represent energy loss (transferring energy to other branches), and negative values represent energy gain. (d) Time evolution of the branch-resolved accumulation of energy transfer from electrons to lattice due to ph-e interactions.

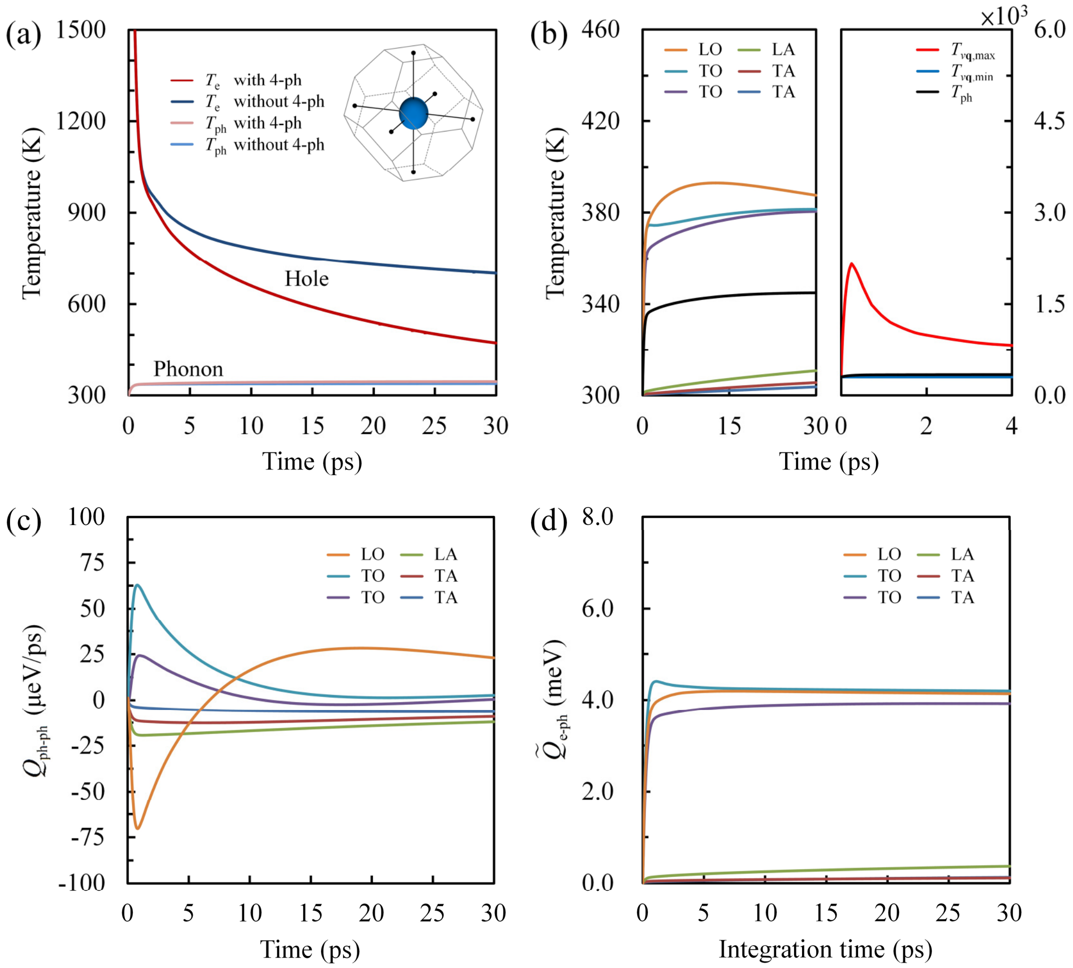

Figure S6. Carrier temperatures and energy transfer of BAs under the *p*-type condition. (a) Time evolution of the temperatures of holes and phonons. (b) Time evolution of the branch-resolved phonon temperatures (left panel); the maximum and minimum temperatures among each phonon mode at each time step (right panel). (c) Time evolution of the branch-resolved phonon energy exchange rate due to ph-ph interactions. Positive values represent energy loss (transferring energy to other branches), and negative values represent energy gain. (d) Time evolution of the branch-resolved accumulation of energy transfer from electrons to lattice due to ph-e interactions.

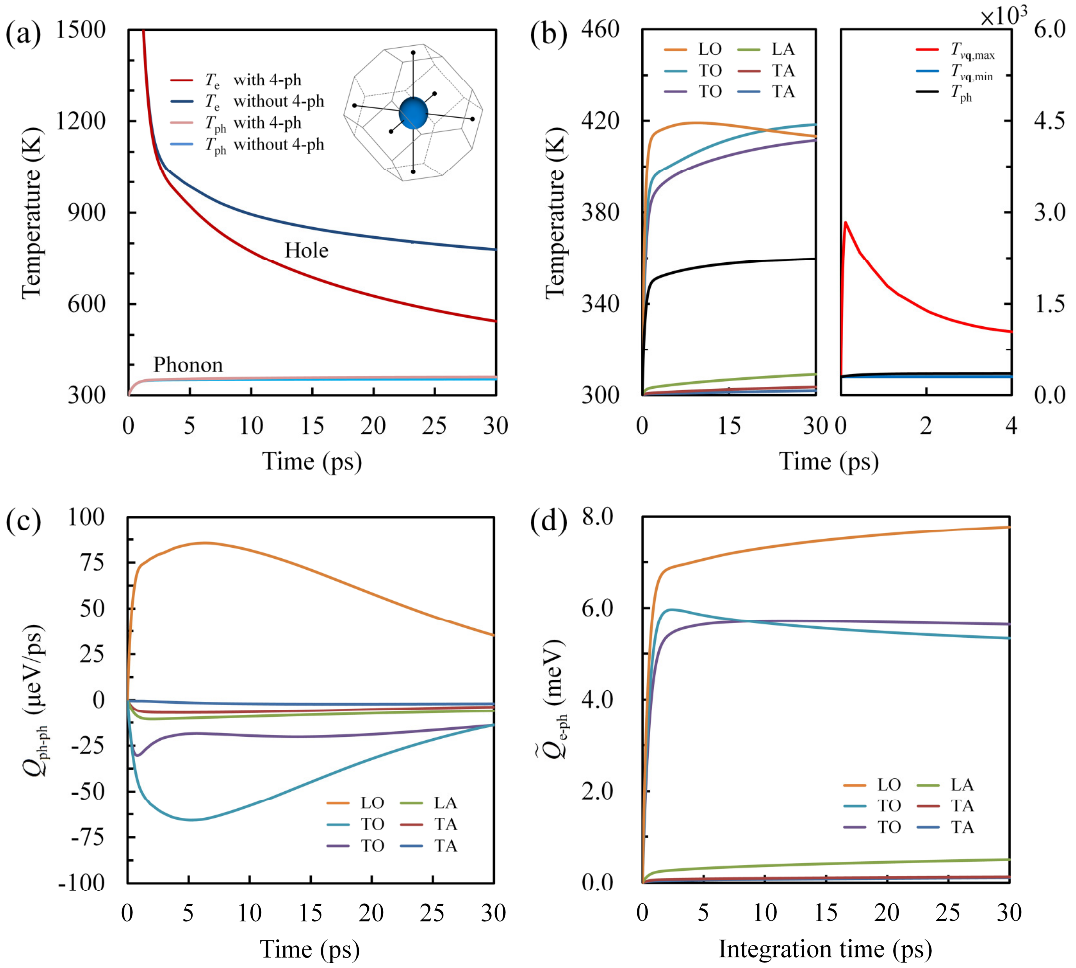

Figure S7. Carrier temperatures and energy transfer of BSb under the *p*-type condition. (a) Time evolution of the temperatures of holes and phonons. (b) Time evolution of the branch-resolved phonon temperatures (left panel); the maximum and minimum temperatures among each phonon mode at each time step (right panel). (c) Time evolution of the branch-resolved phonon energy exchange rate due to ph-ph interactions. Positive values represent energy loss (transferring energy to other branches), and negative values represent energy gain. (d) Time evolution of the branch-resolved accumulation of energy transfer from electrons to lattice due to ph-e interactions.

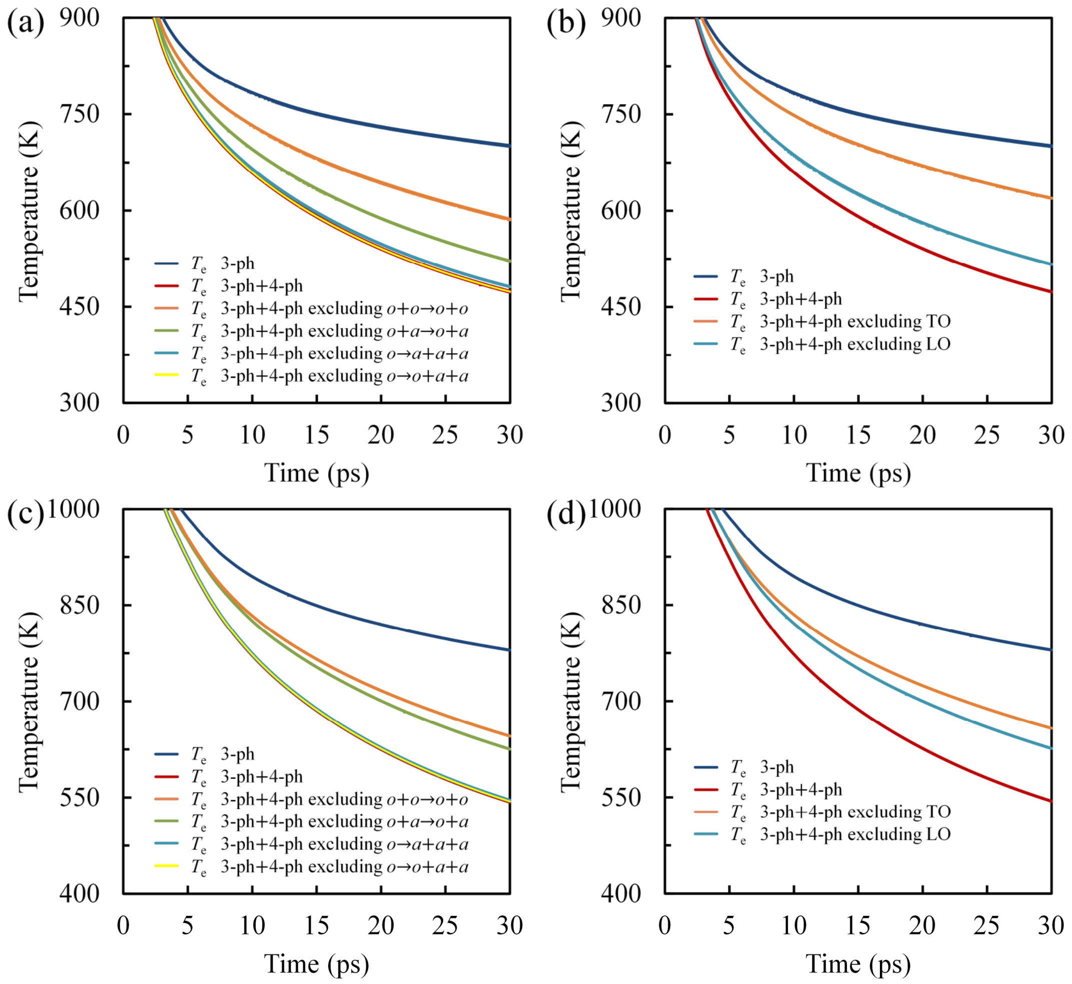

Figure S8. Hole temperatures with selective exclusion of scattering channels in BAs and BSb under the *p*-type condition. (a) Time evolution of hole temperatures of BAs under different four-phonon scattering scenarios. Simulations are performed by selectively excluding the processes-wise scatterings of *o*+*o*→*o*+*o*, *o*+*a*→*o*+*a*, *o*→*a*+*a*+*a*, and *o*→*o*+*a*+*a*, respectively. (b) Time evolution of hole temperatures of BAs with selective exclusion of four-phonon processes involving TO and LO phonons. (c) Time evolution of hole temperatures of BSb under different four-phonon scattering scenarios. (d) Time evolution of hole temperatures of BSb with selective exclusion of four-phonon processes involving TO and LO phonons.

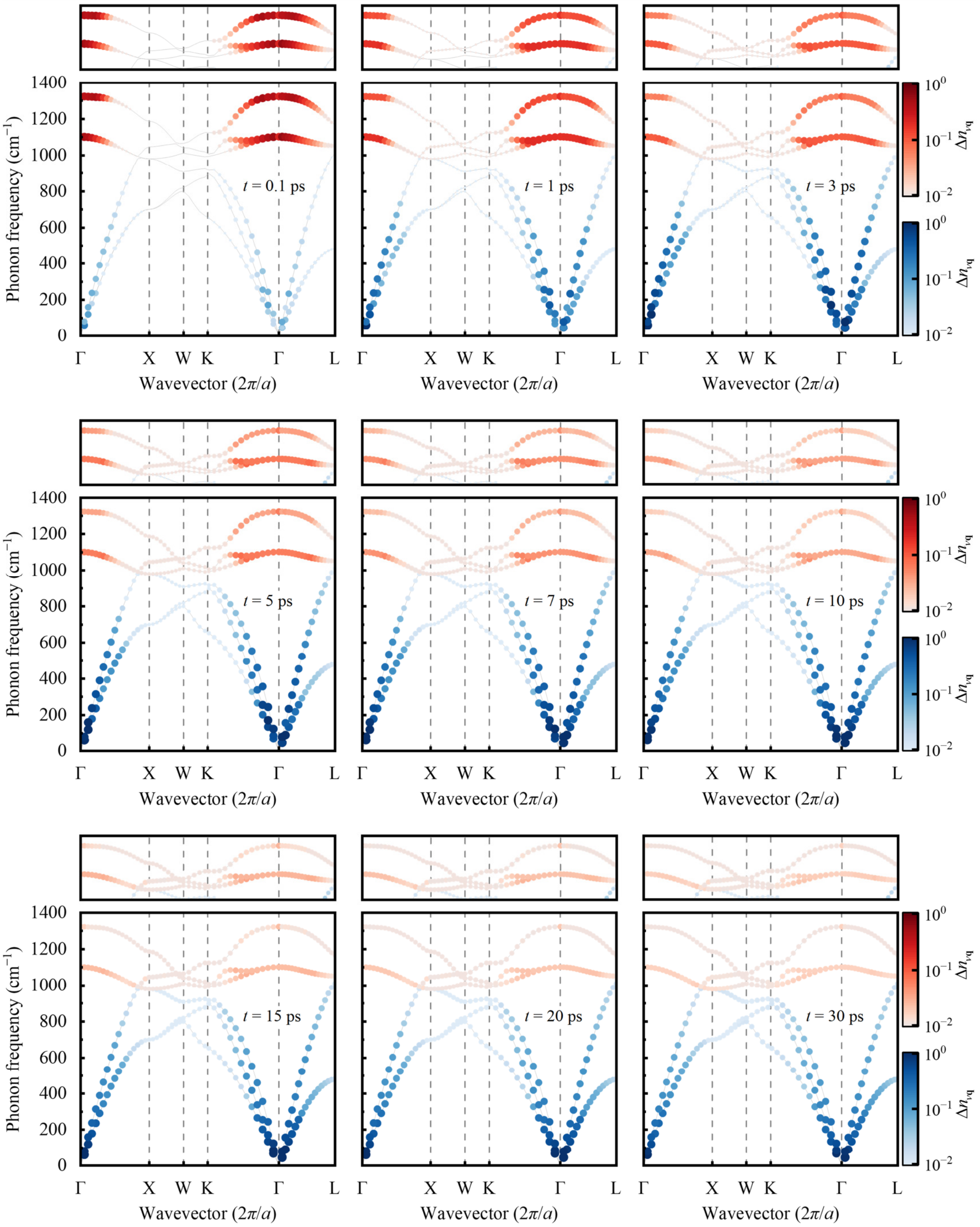

Figure S9. Mode-resolved augmentation of the phonon population of BN under the p-type condition at t = 0.1, 1, 3, 5, 7, 10, 15, 20, and 30 ps. The top panels show results excluding four-phonon couplings. Red and blue denote optical and acoustic phonons, respectively.

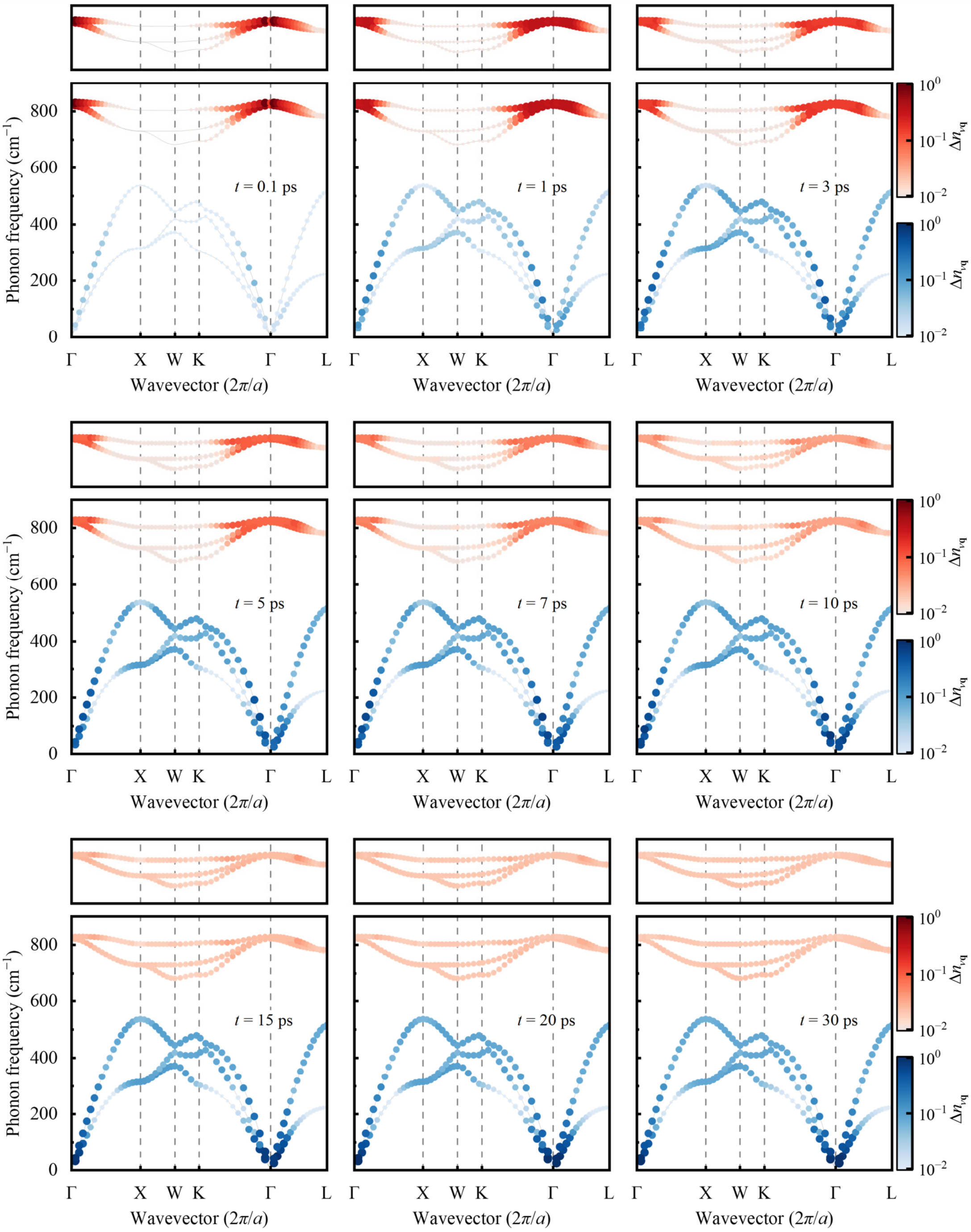

Figure S10. Mode-resolved augmentation of the phonon population of BP under the *p*-type condition at t = 0.1, 1, 3, 5, 7, 10, 15, 20, and 30 ps. The top panels show results excluding four-phonon couplings. Red and blue denote optical and acoustic phonons, respectively.

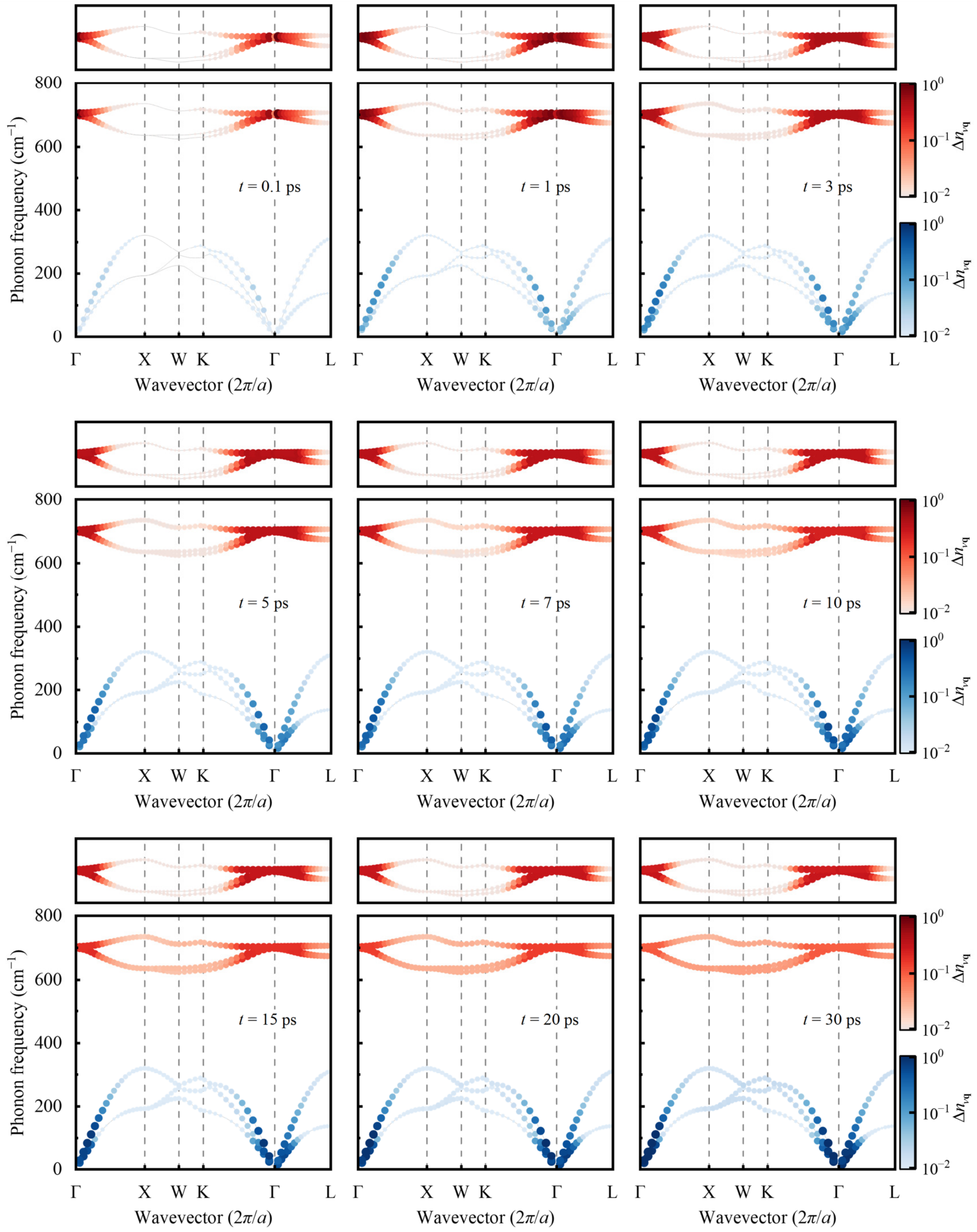

Figure S11. Mode-resolved augmentation of the phonon population of BAs under the *p*-type condition at t = 0.1, 1, 3, 5, 7, 10, 15, 20, and 30 ps. The top panels show results excluding four-phonon couplings. Red and blue denote optical and acoustic phonons, respectively.

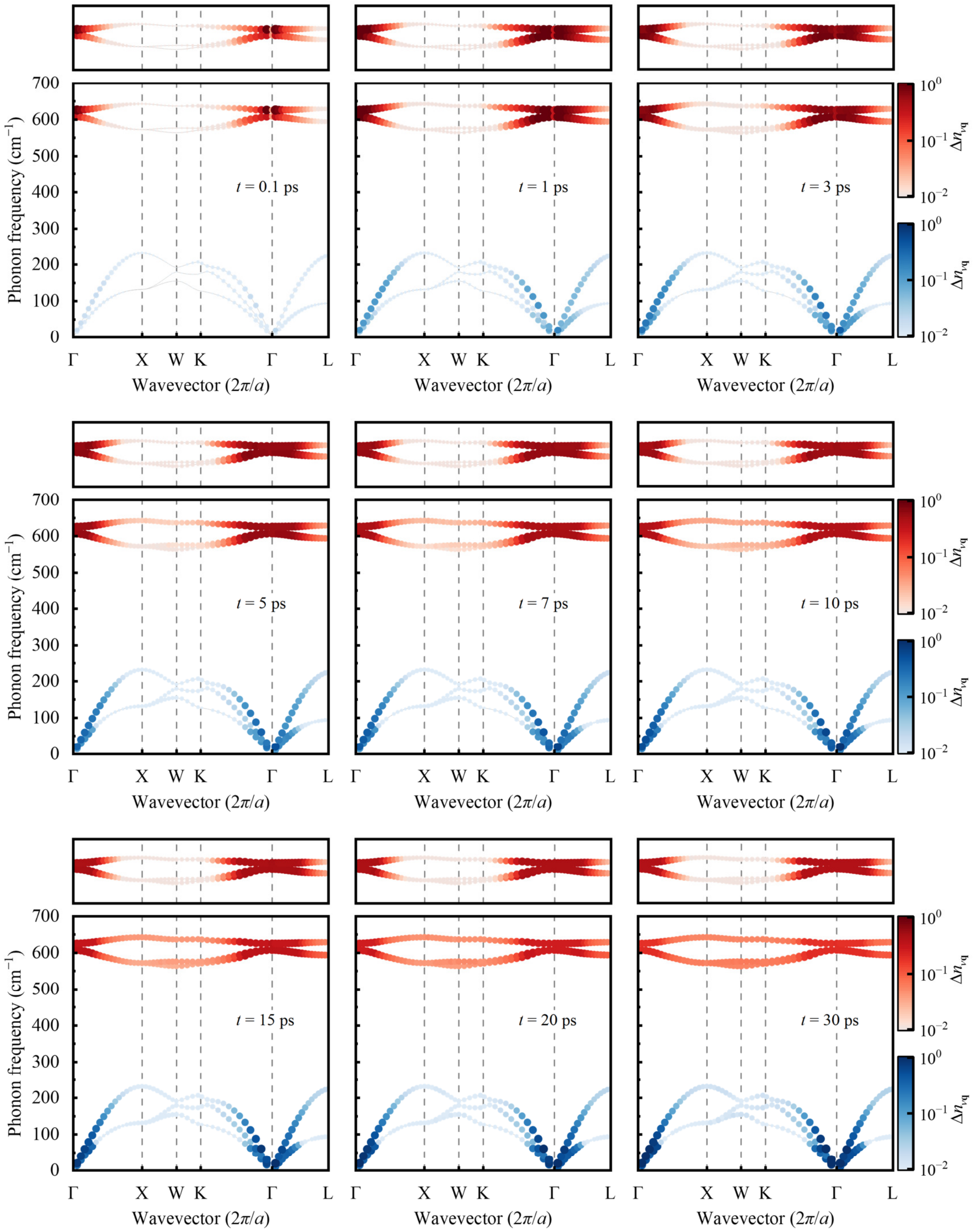

Figure S12. Mode-resolved augmentation of the phonon population of BSb under the *p*-type condition at t = 0.1, 1, 3, 5, 7, 10, 15, 20, and 30 ps. The top panels show results excluding four-phonon couplings. Red and blue denote optical and acoustic phonons, respectively.

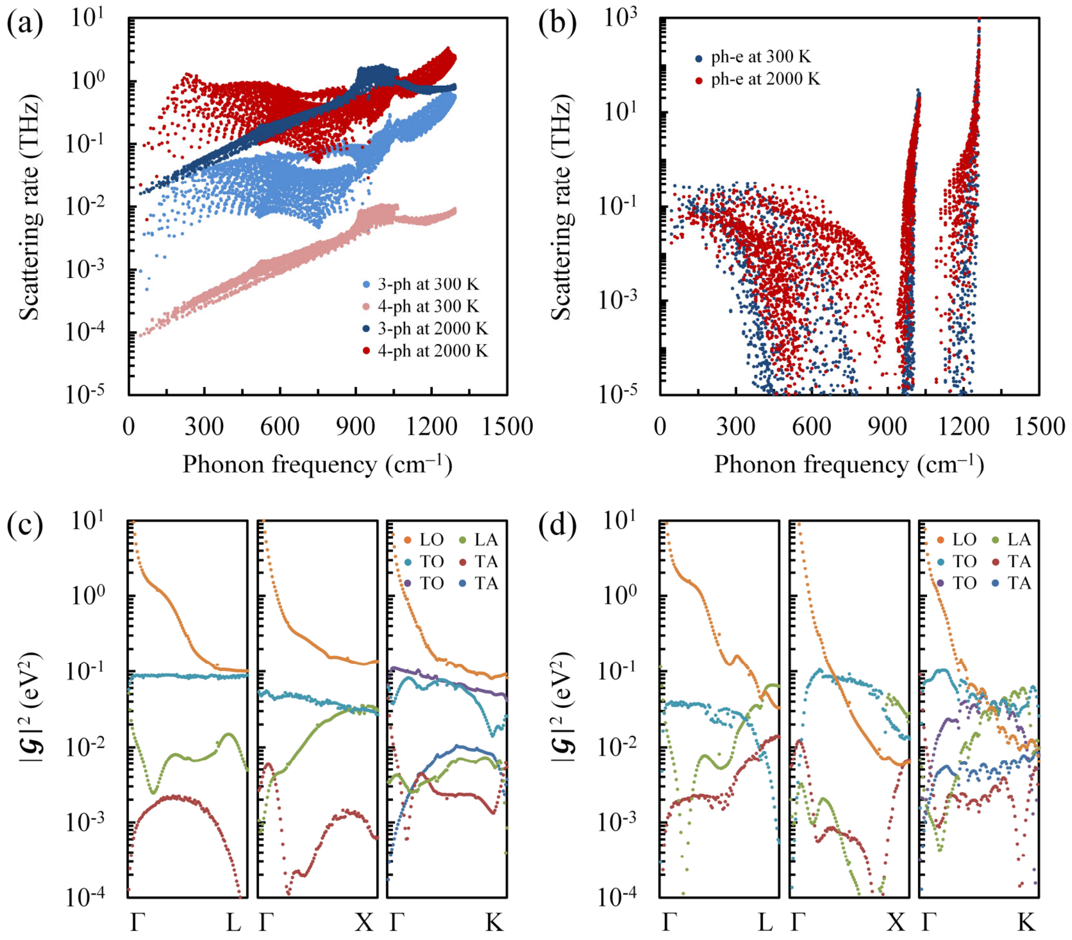

Figure S13. Carrier scattering rates and e-ph coupling matrices of BN under the *p*-type condition. (a) Three- and four-phonon scattering rates at 300 and 2000 K. (b) Phonon-electron scattering rates at 300 and 2000 K. (c) Branch-resolved e-ph coupling matrices between electrons at the valence band maximum with phonons along the three different high-symmetry directions. (d) Branch-resolved e-ph coupling matrices between electrons at the split-off band maximum with phonons along the three different high-symmetry directions.

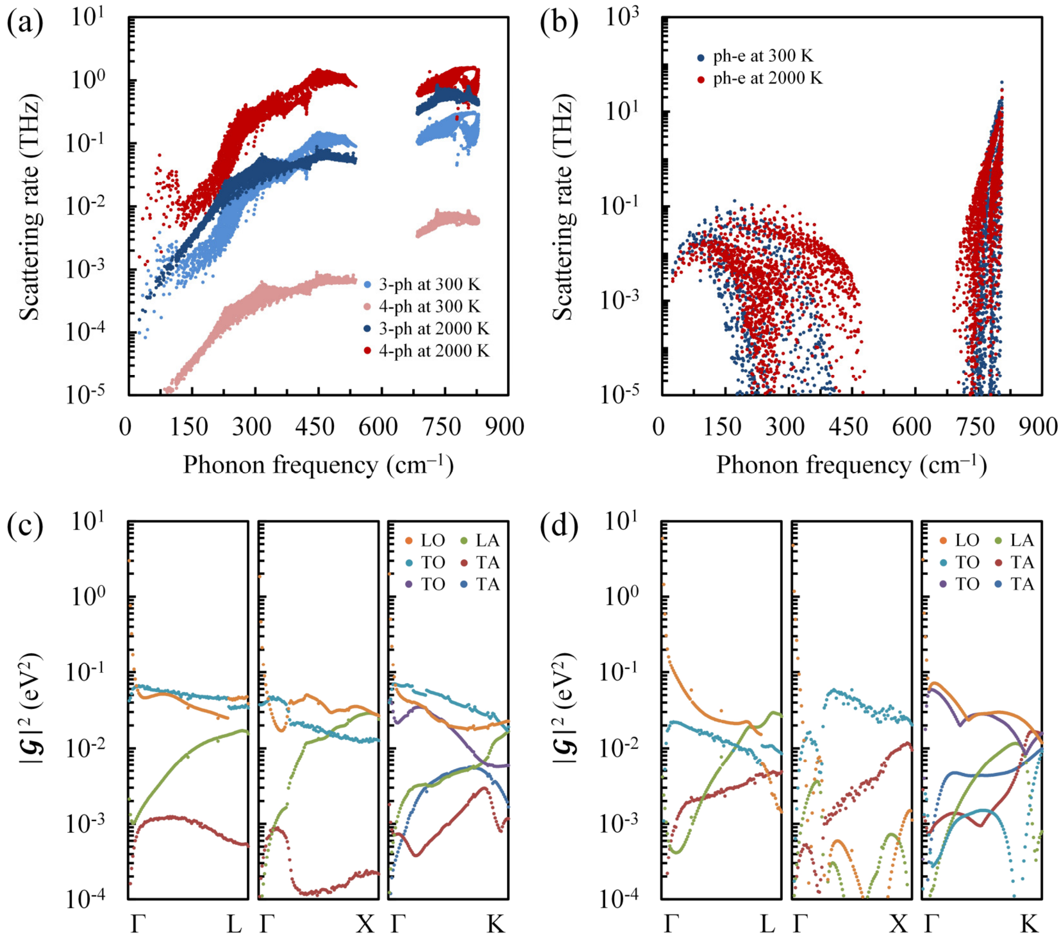

Figure S14. Carrier scattering rates and e-ph coupling matrices of BP under the *p*-type condition. (a) Three- and four-phonon scattering rates at 300 and 2000 K. (b) Phonon-electron scattering rates at 300 and 2000 K. (c) Branch-resolved e-ph coupling matrices between electrons at the valence band maximum with phonons along the three different high-symmetry directions. (d) Branch-resolved e-ph coupling matrices between electrons at the split-off band maximum with phonons along the three different high-symmetry directions.

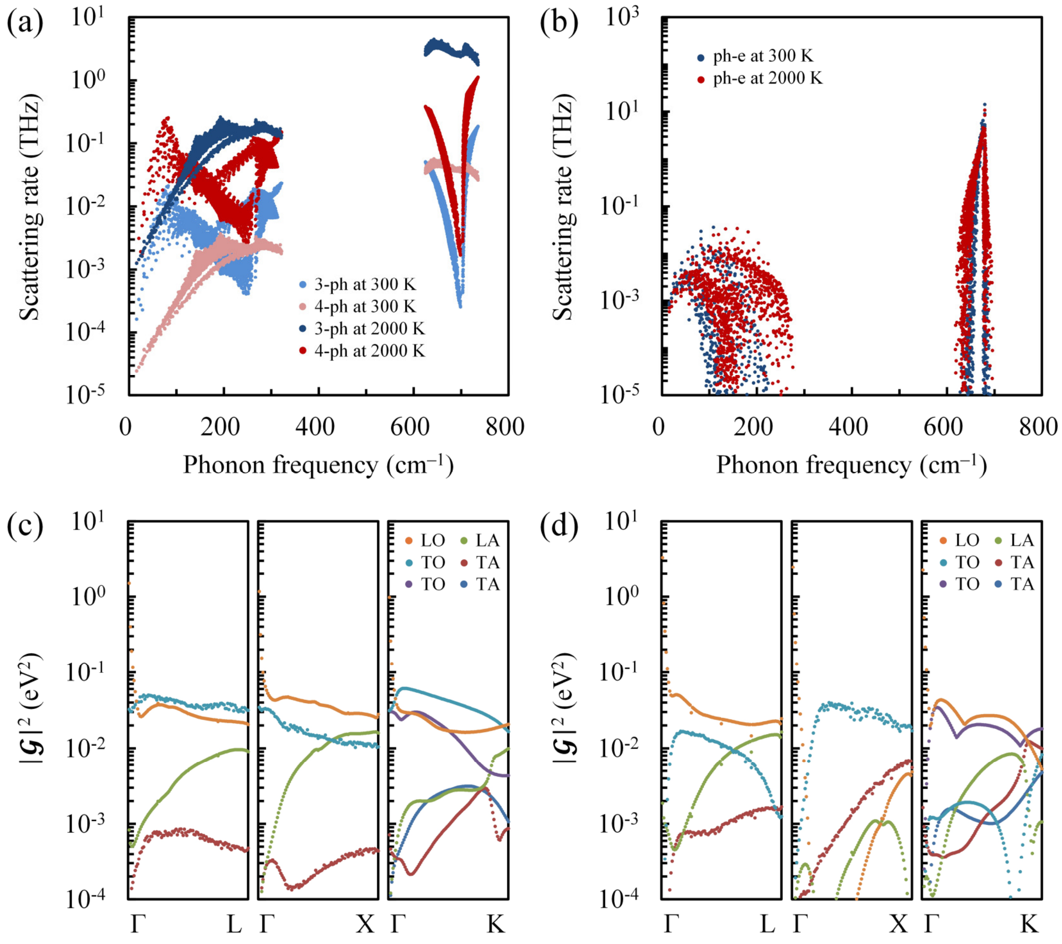

Figure S15. Carrier scattering rates and e-ph coupling matrices of BAs under the *p*-type condition. (a) Three- and four-phonon scattering rates at 300 and 2000 K. (b) Phonon-electron scattering rates at 300 and 2000 K. (c) Branch-resolved e-ph coupling matrices between electrons at the valence band maximum with phonons along the three different high-symmetry directions. (d) Branch-resolved e-ph coupling matrices between electrons at the split-off band maximum with phonons along the three different high-symmetry directions.

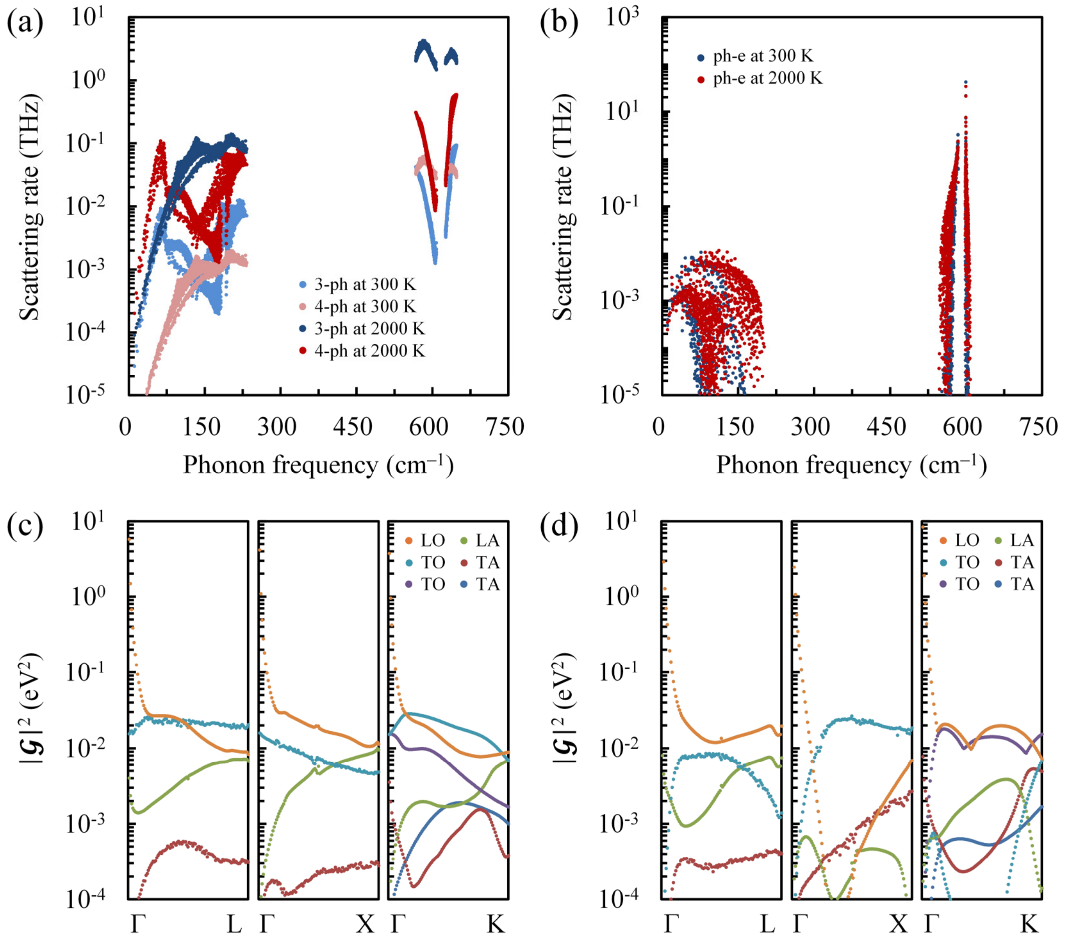

Figure S16. Carrier scattering rates and e-ph coupling matrices of BSb under the *p*-type condition. (a) Three- and four-phonon scattering rates at 300 and 2000 K. (b) Phonon-electron scattering rates at 300 and 2000 K. (c) Branch-resolved e-ph coupling matrices between electrons at the valence band maximum with phonons along the three different high-symmetry directions. (d) Branch-resolved e-ph coupling matrices between electrons at the split-off band maximum with phonons along the three different high-symmetry directions.

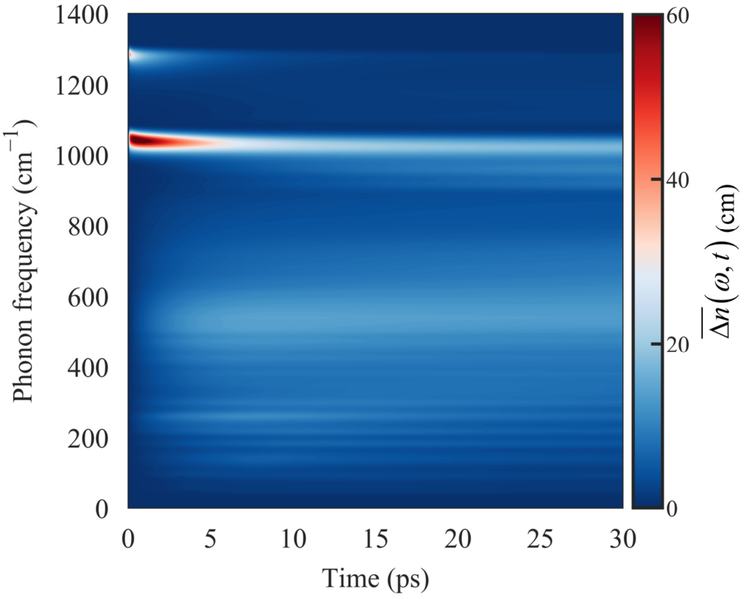

Figure S17. Averaged augmentation of the phonon population of BN under the *p*-type condition. The spectrum is a function of phonon frequency and thermalization time.

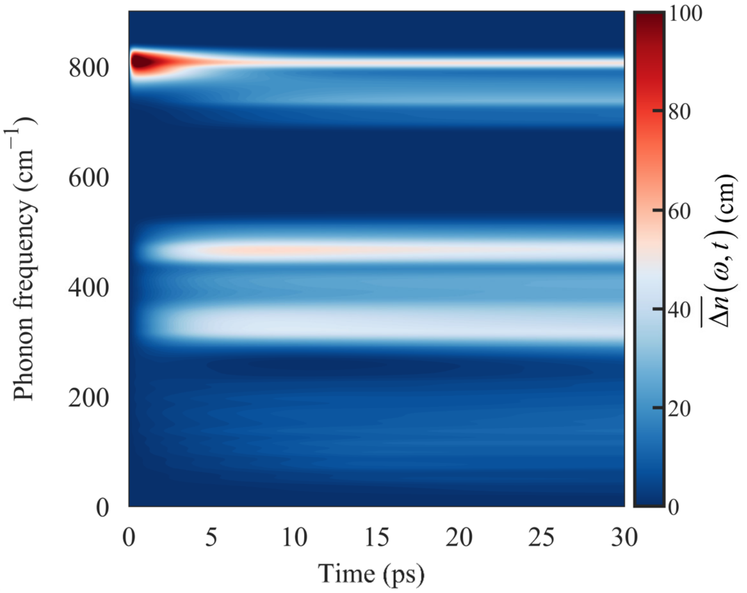

Figure S18. Averaged augmentation of the phonon population of BP under the *p*-type condition. The spectrum is a function of phonon frequency and thermalization time.

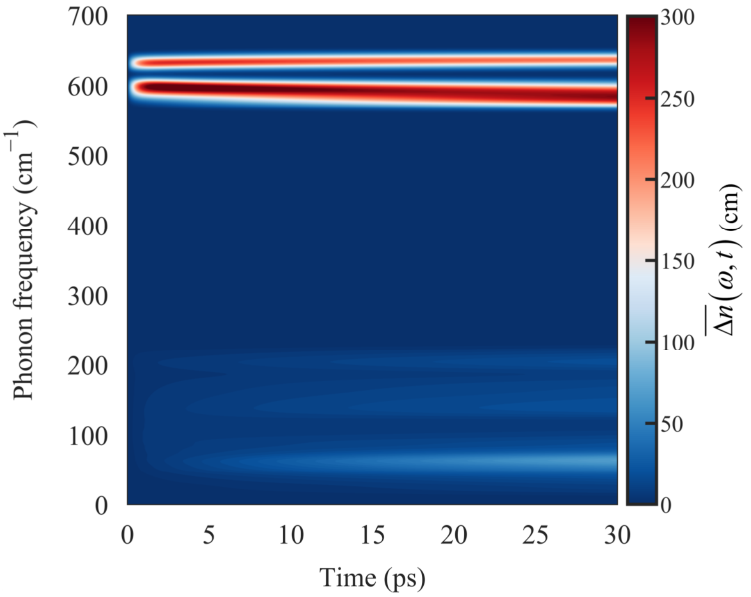

Figure S19. Averaged augmentation of the phonon population of BSb under the *p*-type condition. The spectrum is a function of phonon frequency and thermalization time.

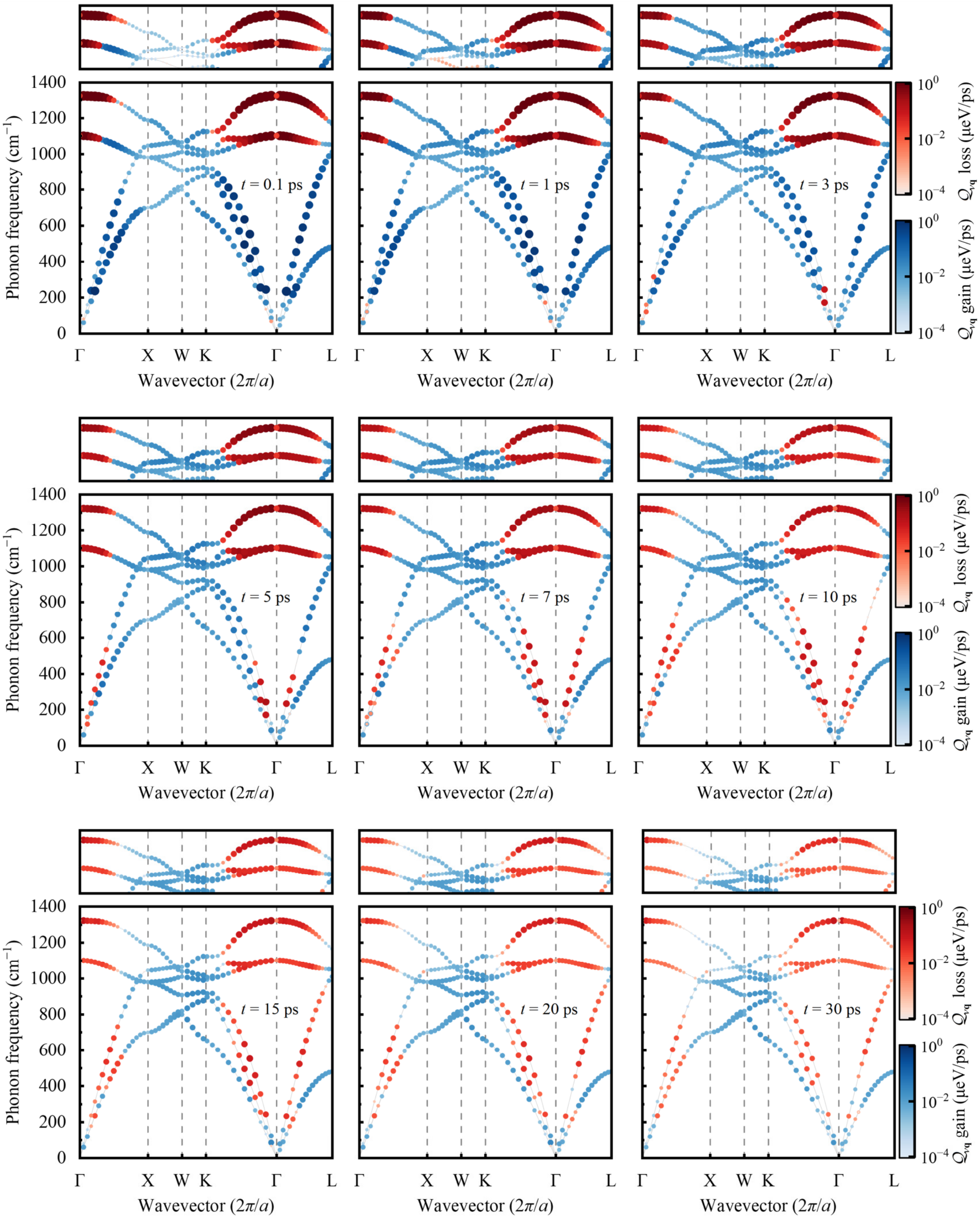

Figure S20. Mode-resolved phonon energy exchange rate due to ph-ph interactions in BN under the p-type condition at t = 0.1, 1, 3, 5, 7, 10, 15, 20, and 30 ps. The top panels show results excluding four-phonon couplings.

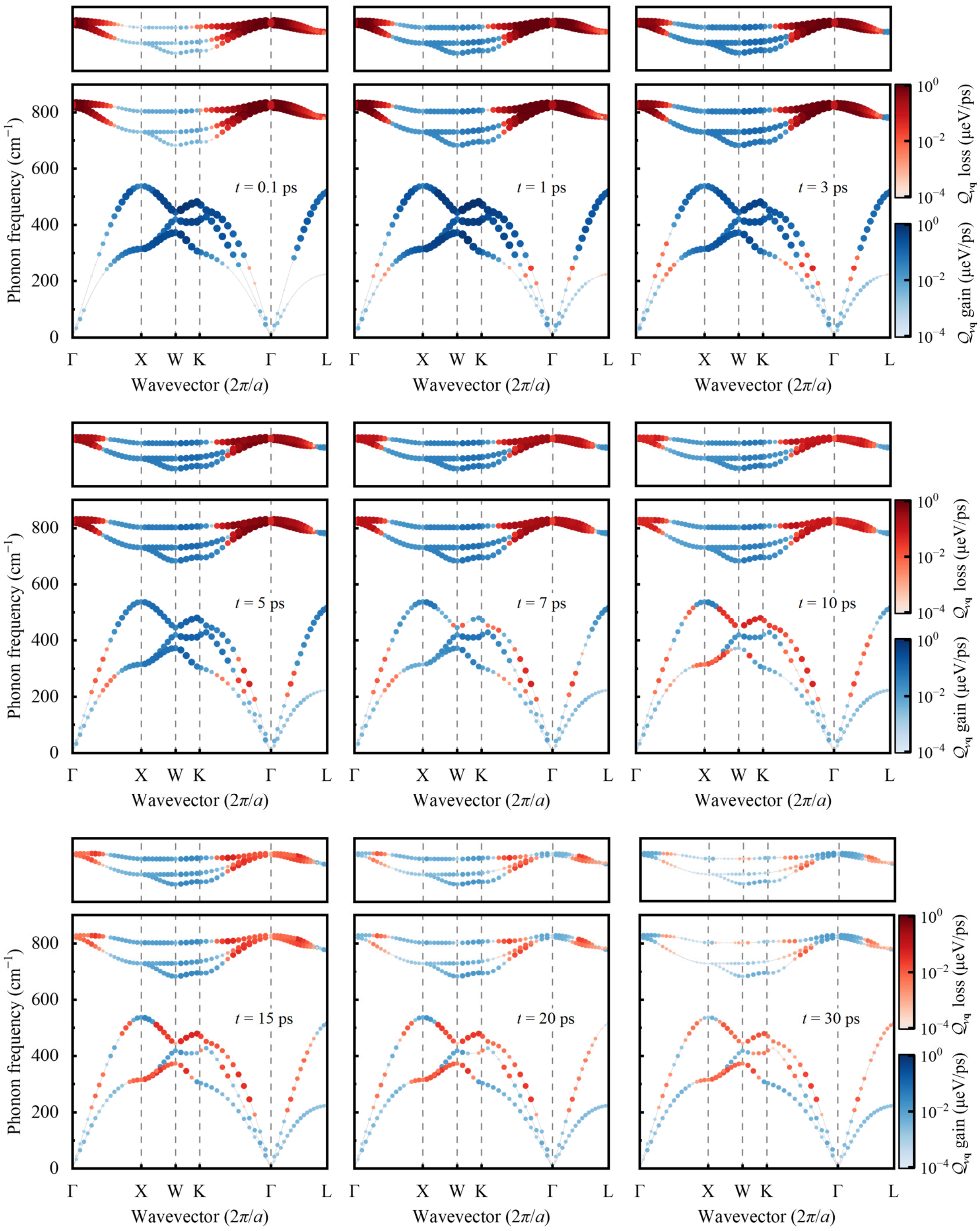

Figure S21. Mode-resolved phonon energy exchange rate due to ph-ph interactions in BP under the p-type condition at t = 0.1, 1, 3, 5, 7, 10, 15, 20, and 30 ps. The top panels show results excluding four-phonon couplings.

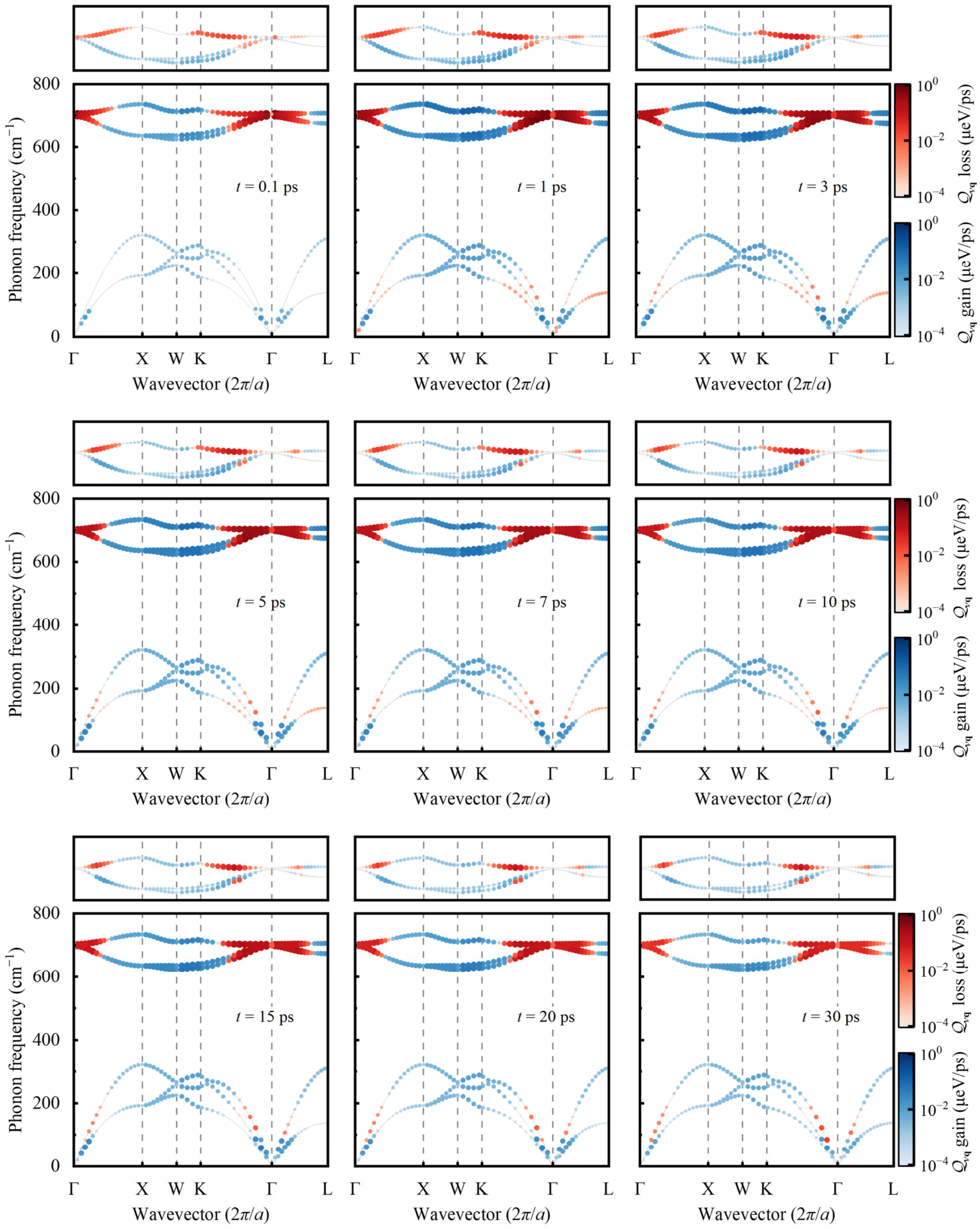

Figure S22. Mode-resolved phonon energy exchange rate due to ph-ph interactions in BAs under the *p*-type condition at t = 0.1, 1, 3, 5, 7, 10, 15, 20, and 30 ps. The top panels show results excluding four-phonon couplings.

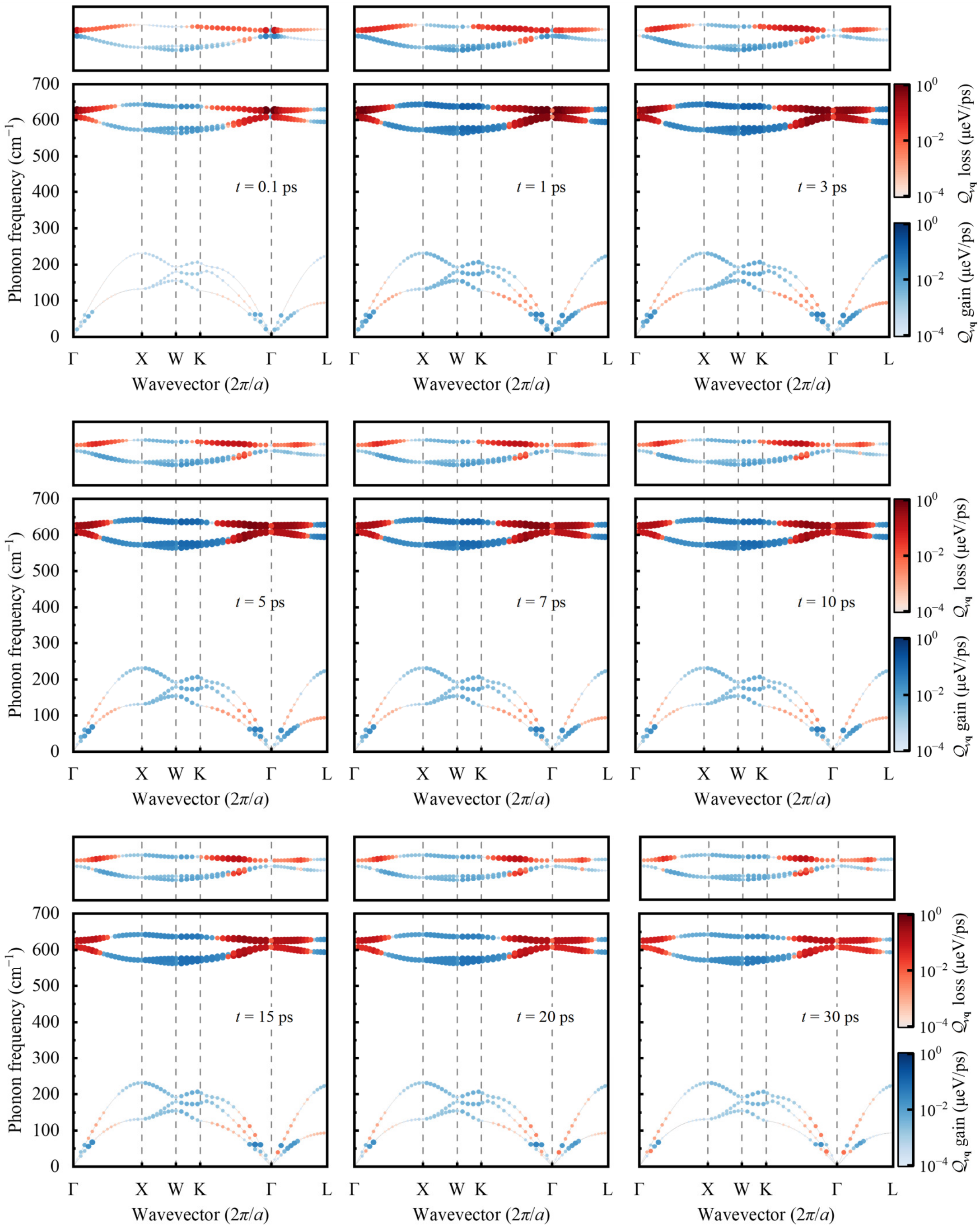

Figure S23. Mode-resolved phonon energy exchange rate due to ph-ph interactions in BSb under the *p*-type condition at *t* = 0.1, 1, 3, 5, 7, 10, 15, 20, and 30 ps. The top panels show results excluding four-phonon couplings.

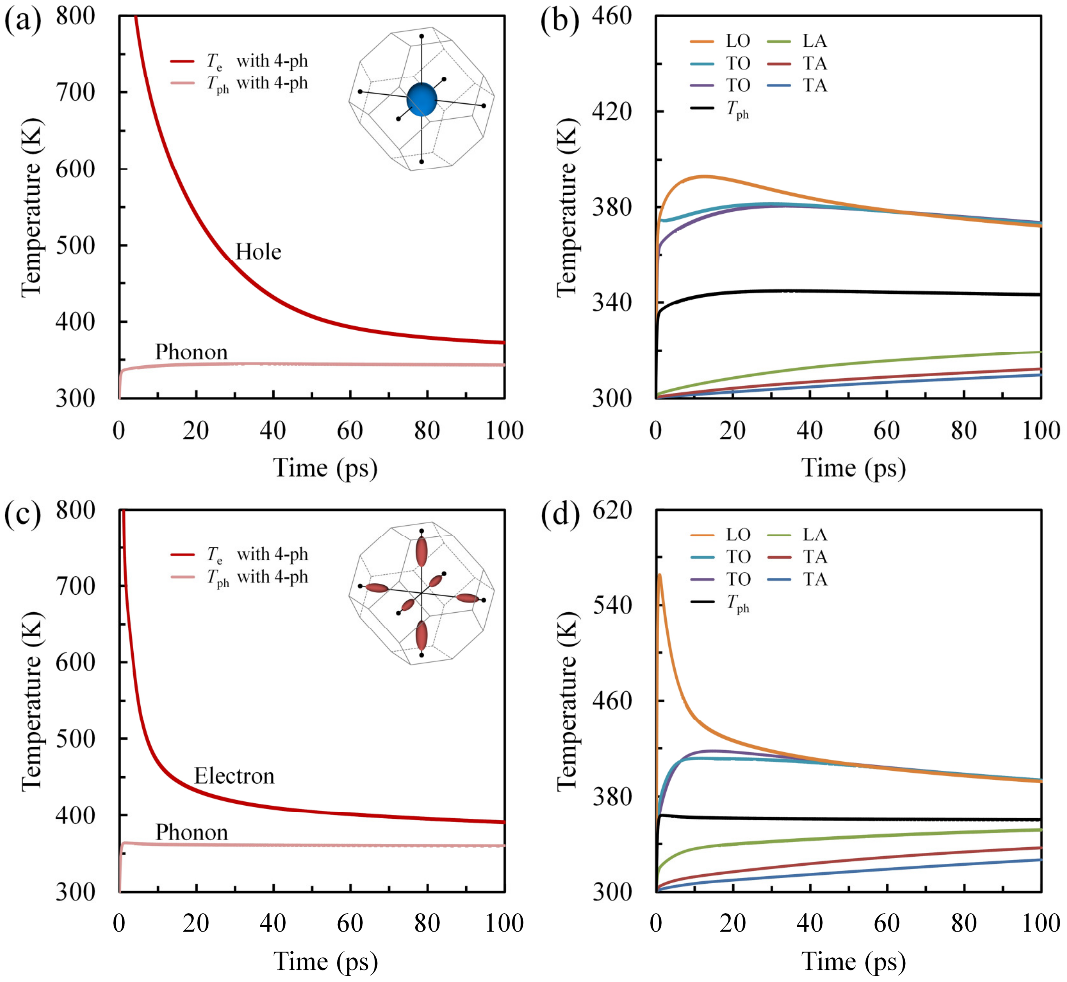

Figure S24. Carrier temperatures of BAs within 100 ps. (a) Time evolution of the temperatures of holes and phonons under the *p*-type condition. (b) Time evolution of the branch-resolved phonon temperatures under the *p*-type condition. (c) Time evolution of the temperatures of electrons and phonons under the *n*-type condition. (d) Time evolution of the branch-resolved phonon temperatures under the *n*-type condition.

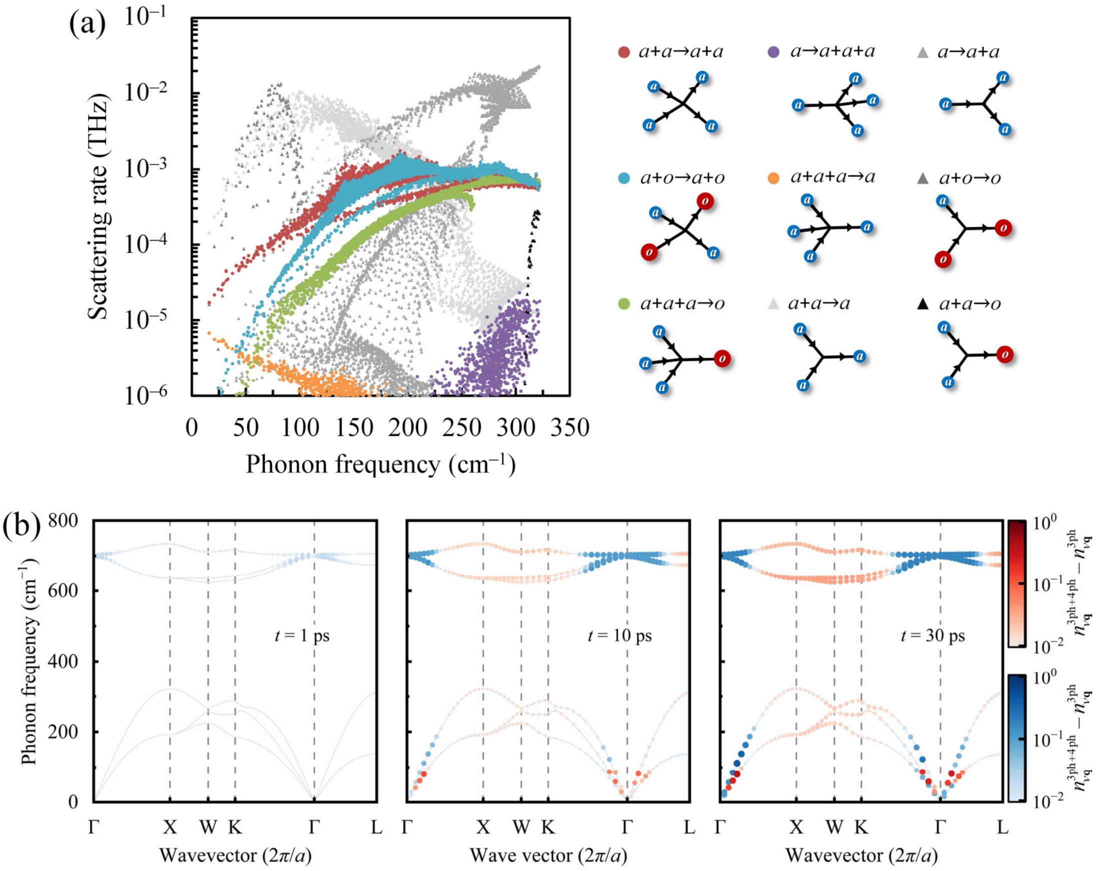

Figure S25. Acoustic-phonon scattering rates and net change of phonon occupation of BAs. (a) Process-wise scattering rates of optical phonons at 300 K. Triangle and circle symbols correspond to results of three- and four-phonon processes, respectively. Schematic illustrations are provided on the right. (b) Mode-resolved net change of the phonon population in BAs under the p-type condition due to four-phonon couplings at t = 1, 10, and 30 ps. Red and blue denote, respectively, an increase and a decrease of phonon population after four-phonon coupling is included.

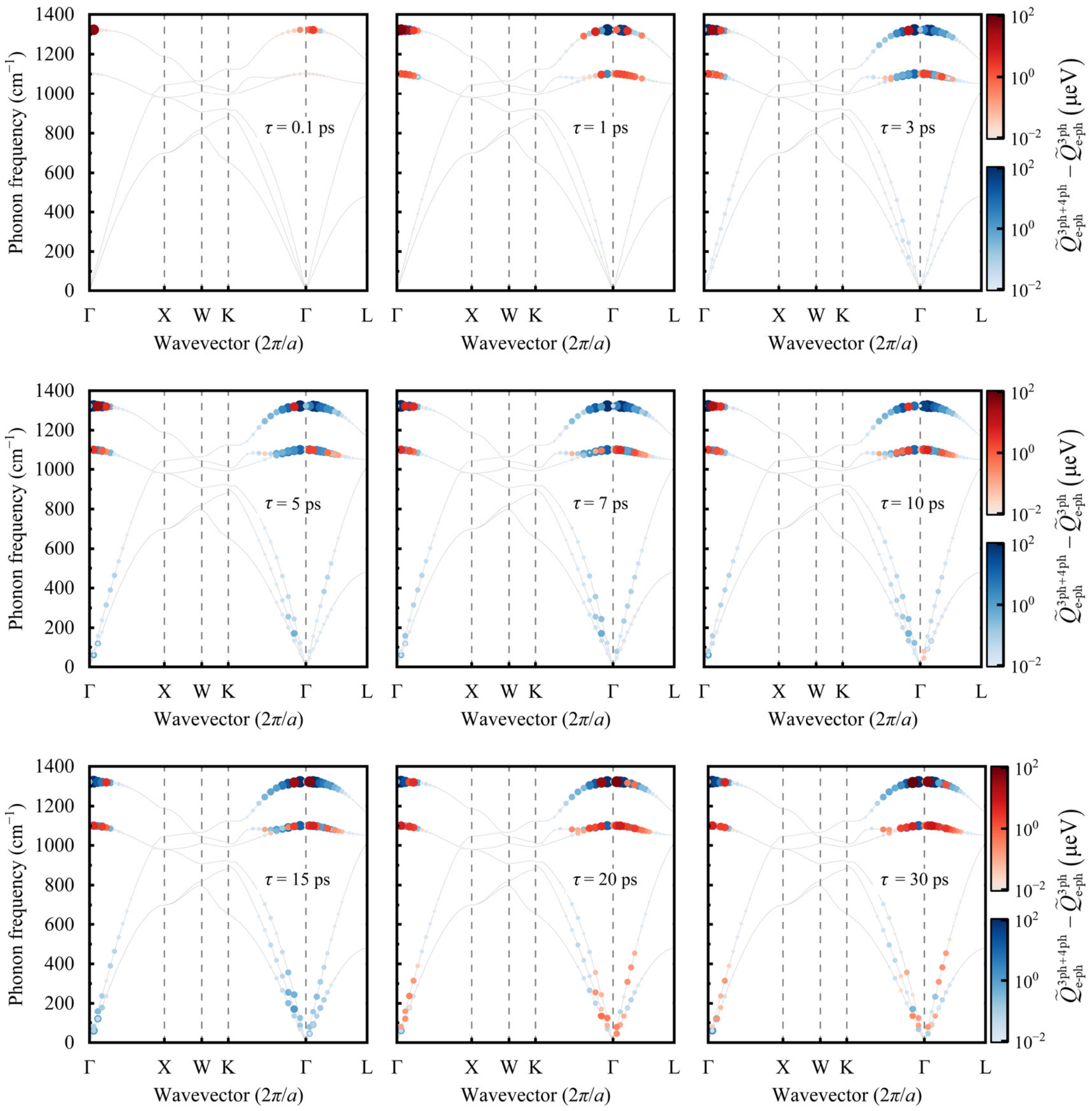

Figure S26. Mode-resolved accumulation of energy transfer from electrons to phonons through e-ph interactions in BN under the *p*-type condition, evaluated at integration times τ = 0.1, 1, 3, 5, 7, 10, 15, 20, and 30 ps. Color symbols show the net change in the energy transfer arising from four-phonon couplings. Red and blue denote an increase and a decrease, respectively.

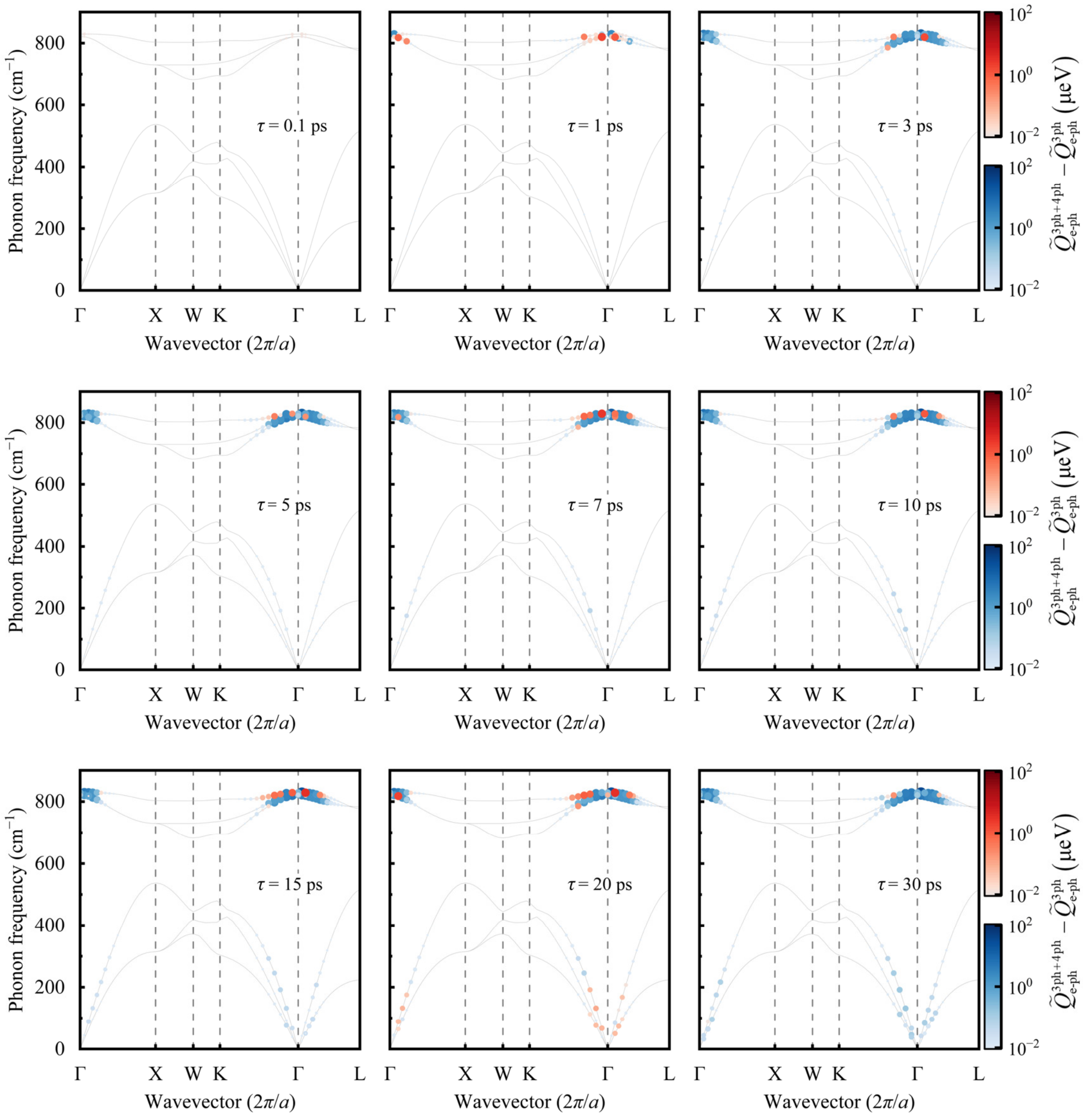

Figure S27. Mode-resolved accumulation of energy transfer from electrons to phonons through e-ph interactions in BP under the *p*-type condition, evaluated at integration times τ = 0.1, 1, 3, 5, 7, 10, 15, 20, and 30 ps. Color symbols show the net change in the energy transfer arising from four-phonon couplings. Red and blue denote an increase and a decrease, respectively.

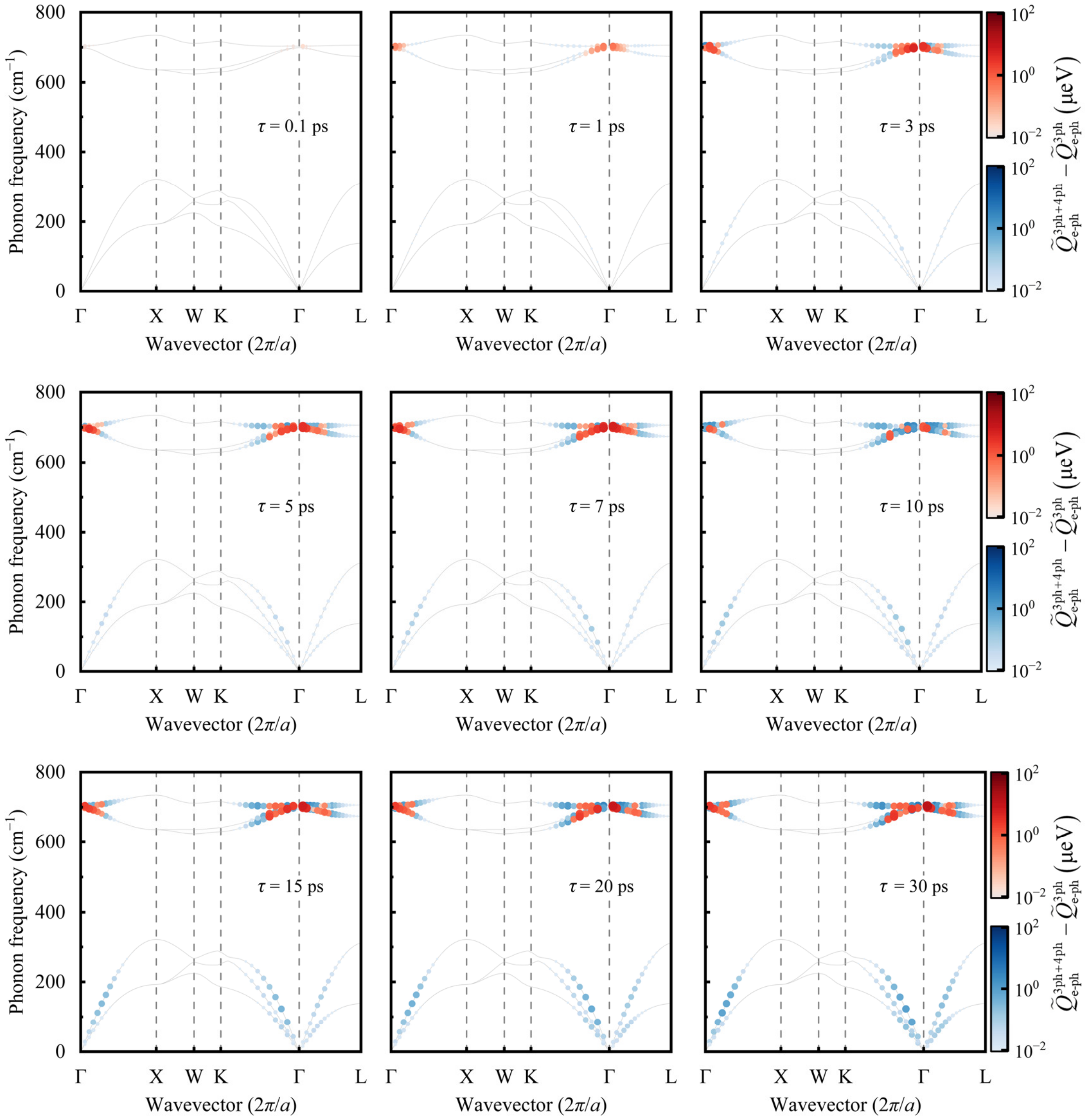

Figure S28. Mode-resolved accumulation of energy transfer from electrons to phonons through e-ph interactions in BAs under the *p*-type condition, evaluated at integration times τ = 0.1, 1, 3, 5, 7, 10, 15, 20, and 30 ps. Color symbols show the net change in the energy transfer arising from four-phonon couplings. Red and blue denote an increase and a decrease, respectively.

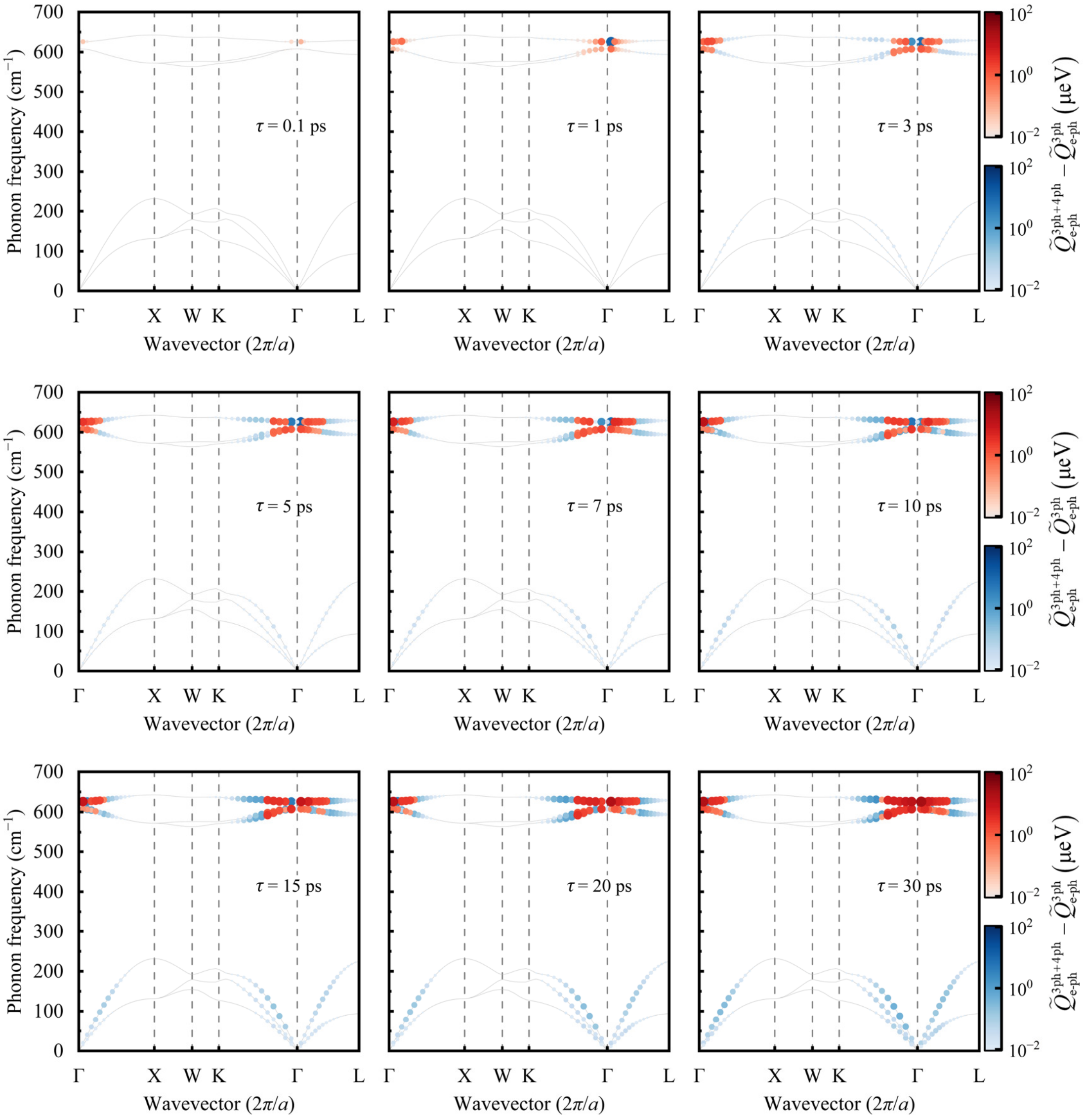

Figure S29. Mode-resolved accumulation of energy transfer from electrons to phonons through e-ph interactions in BSb under the *p*-type condition, evaluated at integration times τ = 0.1, 1, 3, 5, 7, 10, 15, 20, and 30 ps. Color symbols show the net change in the energy transfer arising from four-phonon couplings. Red and blue denote an increase and a decrease, respectively.

Table S1. Bi-exponential fit parameters for the charge-carrier temperature $T_e(t)$ in BP, BAs, and BSb, fitted to $T_e(t) = A_0 + A_1 e^{-t/\tau_1} + A_2 e^{-t/\tau_2}$. Fits are performed for *p*-type conditions within 30 ps with the lattice initially at 300 K and including four-phonon scattering. τ_1 and τ_2 represent the fast (e-ph dominated) and slow (hot-phonon-relaxation dominated) components, respectively.

**Materials**	**Decay constants (ps)**	
	τ_1	τ_2
BP	0.2101	3.4322
BAs	0.3782	11.2647
BSb	0.8381	15.5810

Movie S1. Time evolution of mode-resolved phonon temperatures of BN (top left), BP (top right), BAs (bottom left), and BSb (bottom right). The temperatures of each vibrational state are computed by $T_{\nu\mathbf{q}}(t) = \hbar\omega_{\nu\mathbf{q}}/\left[k_B \ln\left(1/n_{\nu\mathbf{q}}(t) + 1\right)\right]$.

Movie S2. Time evolution of mode-resolved augmentation of the phonon population of BAs. The left panel shows the result including both three- and four-phonon couplings, while the right panel excludes four-phonon couplings.

References

[S1] J. M. Ziman, *Electrons and Phonons: The Theory of Transport Phenomena in Solids* (Oxford University Press, 2001)

[S2] U. Choudhry *et al.*, Persistent hot carrier diffusion in boron arsenide single crystals imaged by ultrafast electron microscopy. *Matter* **6**, 206–216 (2023).

[S3] M. Lundstrom, *Fundamentals of Carrier Transport* (Cambridge University Press, Cambridge, England, 2009).
